# Ratio of n-3/n-6 PUFAs and risk of breast cancer: a meta-analysis of 274135 adult females from 11 independent prospective studies

**DOI:** 10.1186/1471-2407-14-105

**Published:** 2014-02-18

**Authors:** Bo Yang, Xiao-Li Ren, Yuan-Qin Fu, Jin-Long Gao, Duo Li

**Affiliations:** 1 Department of Food Science and Nutrition, Zhejiang University, 866 Yuhangtang Road, Hangzhou 310058, China; 2 Department of Preventive Medicine, Wenzhou Medical University, Wenzhou, China; 3 Medical Laboratory Animal Center, Wenzhou Medical University, Wenzhou, China

## Abstract

**Background:**

Increased ratio of n-3/n-6 polyunsaturated fatty acids (PUFAs) in diet or serum may have a protective effect on the risk of breast cancer (BC); however, the conclusions from prospective studies are still controversial. The purpose of this study is to ascertain the relationship between intake ratio of n-3/n-6 PUFAs and the risk of BC, and estimate the potential summarized dose–response trend.

**Methods:**

Relevant English-language studies were identified through Cochrane Library, PubMed and EMBASE database till April 2013. Eligible prospective studies reporting the multivariate adjusted risk ratios (RRs) for association of n-3/n-6 PUFAs ratio in diet or serum with BC risk. Data extraction was conducted independently by 2 investigators; disagreements were reconciled by consensus. Study quality was assessed using the Newcastle-Ottawa scale. Study-specific RRs were combined via a random-effects model.

**Results:**

Six prospective nested case–control and 5 cohort studies, involving 8,331 BC events from 274,135 adult females across different countries, were included in present study. Subjects with higher dietary intake ratio of n-3/n-6 PUFAs have a significantly lower risk of BC among study populations (pooled RR = 0.90; 95% CI: 0.82, 0.99), and per 1/10 increment of ratio in diet was associated with a 6% reduction of BC risk (pooled RR = 0.94; 95% CI: 0.90, 0.99; *P* for linear trend = 0.012). USA subjects with higher ratio of n-3/n-6 in serum phospholipids (PL) have a significantly lower risk of BC (pooled RR = 0.62; 95% CI: 0.39, 0.97; *I*^*2*^ = 0.00%; *P* for metaregression = 0.103; *P* for a permutation test = 0.100), and per 1/10 increment of ratio in serum PL was associated with 27% reduction of BC risk (pooled RR = 0.73; 95% CI: 0.59, 0.91; *P* for linear trend = 0.004; *P* for metaregression = 0.082; *P* for a permutation test = 0.116).

**Conclusions:**

Higher intake ratio of n-3/n-6 PUFAs is associated with lower risk of BC among females, which implies an important evidence for BC prevention and treatment is by increasing dietary intake ratio of n-3/n-6 PUFA. No firm conclusions from USA populations could be obtained, due to the limited numbers of USA studies.

## Background

Although breast cancer (BC) is the most common cancer occurring among women worldwide, international variation of BC incidence show there is a higher incidence in North America and Western Europe, but lower incidence in Asia [1,2]. The large geographic heterogeneity of incidence among women globally could be explained by variation of dietary patterns, especially with relation to dietary fat as a potential dietary factor that is closely correlated with increased incidence of BC [3-6]. Polyunsaturated fatty acids (PUFAs) as dietary fat subtypes consist of two families: n-3 PUFAs and n-6 PUFAs. Serum phospholipids (PL) ratio of n-3/n-6 PUFA can directly reflect dietary intake ratio of n-3/n-6 PUFA, due to the lack of interconversion between n-3 and n-6 PUFAs in humans. N-3 and n-6 PUFAs *in vivo* can influence breast tumor cell growth by simultaneously competing for the same metabolic pathway (COX and LOX pathway) to change the balance of tissue eicosanoids, the transcription mediated by nuclear factor κ*B* (NF-κB), and signal transduction mediated by the mammalian target of rapamycin (mTOR) etc. [7-9]. Therefore, ratio of n-3/n-6 PUFAs in diet and serum PL probably plays an important role in the risk of BC.

The studies from cell lines and animals have shown promising results of down regulating BC tumor growth by n-3 PUFAs as a nutrient to compete with n-6 PUFAs [10,11]. Most of the case–control studies also support that dietary or serum PL n-3/n-6 ratio is inversely associated with risk of BC [4,5,12-14]. However, there are some inconsistent conclusions in prospective studies [15-20], and the optimal intake ratio of n-3/n-6 PUFAs has not yet been well defined. Therefore, it is necessary to quantitatively ascertain the association between intake ratio of n-3/n-6 PUFAs and the risk of BC by means of meta-analysis. Available data from prospective studies of adult females (premenopausal, postmenopausal, or combined) across different countries were pooled to summarize the relationship between intake ratio of n-3/n-6 PUFAs and the risk of BC for highest vs. lowest quantile, to estimate the potential dose–response trend and to conduct the stratified analysis for exploring the probable source of heterogeneity.

## Methods

### Literature search

We identified prospective studies which reported the association between intake ratio of n-3/n-6 (n-6/n-3) and BC risk up until April 2013 from PubMed, Embase, and Cochrane Library database using literature retrieval of subject headings. Search strategy was (*“Fatty Acids, Omega-3″OR “Fatty Acids, Omega-6″) AND “Breast Neoplasms”* for PubMed, *“Breast tumor” AND* (*“omega 3 fatty acid” OR “omega 6 fatty acid”)* for EMBASE and *“Fatty Acids”AND “Breast Neoplasms” * for Cochrane Library databases. We also searched systematic reviews from the above-mentioned database, and checked reference lists to identify studies that might have been missed. The present meta-analysis was conducted using the standard methods from Cochrane Collaboration, and reporting items were mainly based on MOOSE guidelines for meta-analysis of observational studies [21] (Additional file 1). Ethical approval and informed consent were not required for this meta-analysis.

### Eligibility criteria

1) Participants: study population included any adult women (premenopausal, postmenopausal, or combined), whose base conditions were regarded as stable; 2) Exposure: evaluating ratio of n-3/n-6 (n-6/n-3) PUFAs in diet or human serum (plasma) PL; 3) Outcomes: evaluating BC incidence as outcome variable and providing risk ratios (RRs) for all categories of dietary or serum (plasma) PL ratio of n-3/n-6 or n-6/n-3 PUFAs; 4) Study Design: prospective studies (cohort, nested case–control and case-cohort study) were included.

### Study identification

Two trained investigators (YF and JG) identified articles eligible for further review by performing a stepwise screening of titles or abstracts, followed by a full-text review based on common inclusion criterion. Discrepancies were resolved through discussion with the third investigator (BY). Studies of cross-sectional, cross-over, randomized controlled trials (RCT), experimental designs (cell culture and animal test), non- original research (reviews, editorials, or commentaries), abstract, unpublished studies, or duplicated studies were excluded. Our search was restricted to human studies published in English. We did not contact authors for the detailed information of primary studies only reporting association of n-3 or n-6 PUFA with BC risk. We contacted the authors of the two studies reporting association of n-3 and n-6 PUFA with BC risk by email.

### Data extraction and quality assessment

Data extraction was finished independently and performed twice by two reviewers (YF and JG), and disagreements were reconciled by consensus. Detailed data concerning participants, exposure, comparability and outcomes were extracted using a standard extraction form (Additional file 2). We mainly aimed to extract the characteristics of participants (e.g., nationality, age, menopausal status, follow-up duration and number of participants), intake n-3/n-6 (n-6/n-3) ratio exposure (e.g., measurement method, exposure source, and exposure range), covariates adjusted in multivariable analysis and RRs including corresponding confidence intervals (CIs) for all categories of dietary or serum (plasma) PL ratio of n-3/n-6 (n-6/n-3) PUFAs. Quality assessment was performed by using the Newcastle-Ottawa scale (NOS) [22], which mainly contains selection domain (0–4 stars), comparability domain (0–2 stars) and exposure or outcomes domain (0–3 stars).

### Data synthesis and statistic analysis

In this meta-analysis, intake ratio of n-3/n-6 PUFAs was defined as the proportion of total n-3 PUFAs (the sum of ALA, EPA and DHA) to total n-6 PUFAs in diet or serum (plasma) PL, and pooled RR including corresponding 95% CI was taken as the summary risk estimate for all studies. RRs from each study were firstly transformed to their logarithm (logRR), and corresponding 95% CIs were used to calculate corresponding standard errors (selogRR). We conducted two types of meta-analysis. Firstly, we conducted meta-analysis for the highest quantile (tertile, quartile and quintile) compared with lowest or reference, and study-specific RRs were combined using a random-effects model described by DerSimonian and Laird [23], which considers both within-study and between-study variability. Subsequently, summary dose–response meta-analysis was performed using the method described by Greenland and Orsini, et al. [24,25] to estimate the potential linear trend and achieve association between per 1/10 increment of intake ratio of n-3/n-6 PUFAs and BC risk (Additional file 3). To examine a potential nonlinear (curvilinear) trend, we used the restricted cubic splines functional model with three knots at percentiles 25%, 50%, and 75% of the distribution. A p-value for curvilinear trend was calculated by testing the null hypothesis that the coefficient of the second spline is equal to zero [26,27].

Heterogeneity was assessed with the *Q* test and *I*^*2*^ statistic. We considered an *I*^*2*^ value greater than 50% and 2-tailed *P* < 0.10 as indicative of heterogeneity according to Cochrane Handbook, and defined the low, moderate and high degrees of heterogeneity by *I*^*2*^ values of 25%, 50% and 75% as cut-off points [28] respectively. If heterogeneity was presented in this meta-analysis, metaregression and subgroup analyses were conducted to identify the potential sources of heterogeneity by study design (cohort study and nested case–control study), different regions (Europe, USA and Asia), menopausal status (pre-, post- and combined), and follow-up duration (more than and less than average value) and known covariates adjusted (e.g., BMI, age, and family history of BC) in multivariate analysis.

Sensitivity analysis was performed to evaluate potential influence of individual study on overall risk estimation, and compare the pooled RR from random effect model with that from fixed effect model. Potential publication bias was qualitatively delineated by the asymmetry of funnel plot, and it was also quantitatively examined by Begg’s test and Egger’s regression test (*P* < 0.05 was considered representative of statistical significance) [29]. If potential publication bias was found in the meta-analyses, contour-enhanced funnel plot was performed to explore the probable source of publication bias [30]. This method examined the visual asymmetry of funnel plot, and differentiated asymmetry due to publication bias from other factors [31]. Statistical analysis of the combined data was performed by STATA version 11.0 (Stata CORP, College Station, TX).

## Results

**Figure 1:**
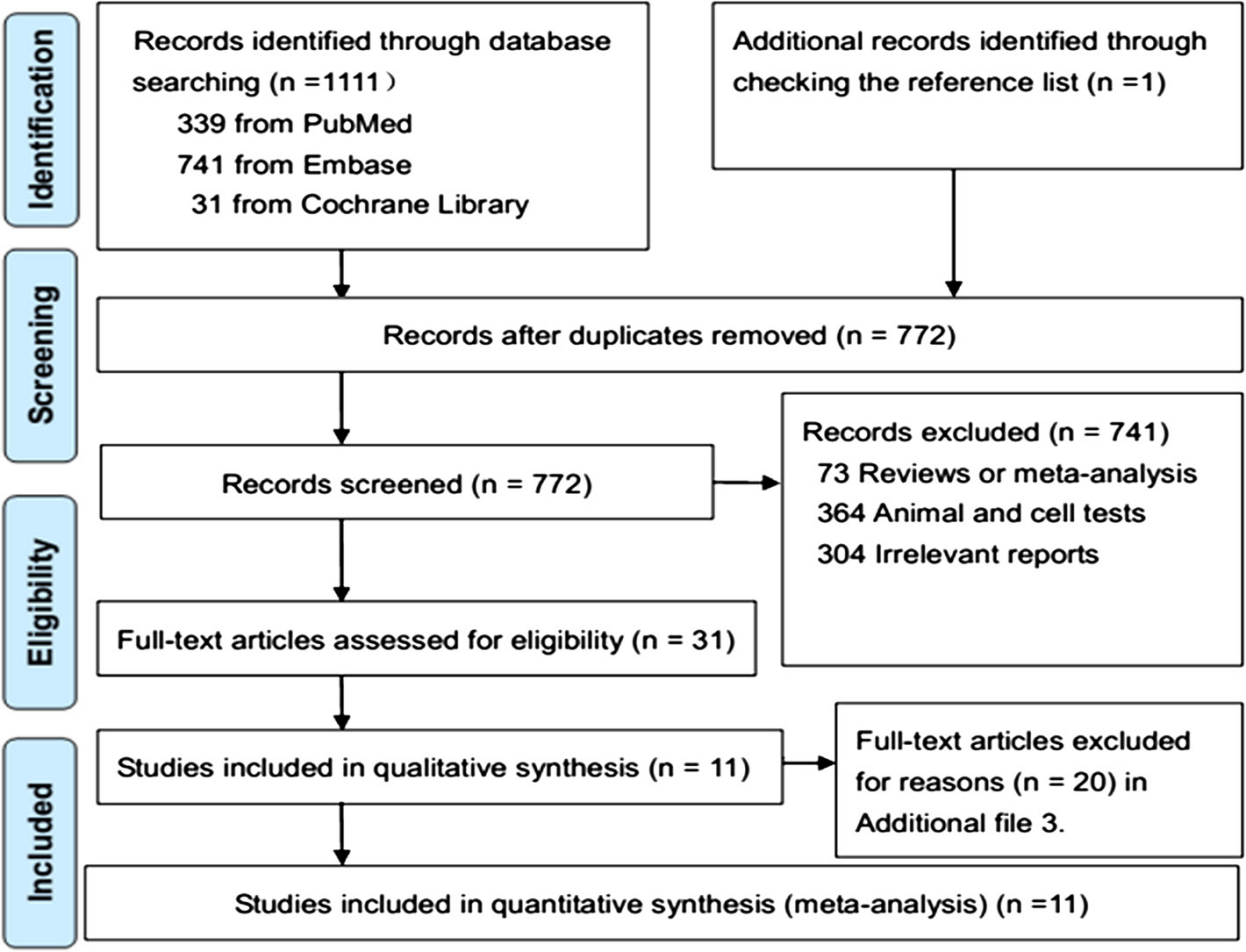
PRISMA flow diagram for included prospective studies.

We identified 1,112 potential studies from electronic search, and 772 studies were left after removing duplicates. Thirty one prospective studies were obtained after title and abstract review. Eleven studies were eligible for inclusion in the present study after full text review (Figure 1), and 20 studies were excluded for other reasons (Additional file 3).

### Characteristics of the included studies

Included studies consist of 5 prospective cohort studies [15-17,32,33] and 6 prospective nested case–control studies [18-20,34-36] (Table 1; Additional file 1). The 6 studies looked at intake of dietary fatty acid [15-17,32,33,35], which was quantified by food frequency questionnaires using the measurement grams per day (g/d) [16,33,35], percentage of energy (% energy) [17,32], and grams per 1,000 kilocalorie (g/1000 kcal) [15]. There are 5 studies concerning serum PL biomarker, where fatty acid compositions in serum PL was quantified by gas chromatography, and measurement unit was percentage of total fatty acids, except for 1 study (mg/L) [18]. One study provided data of pre- and post-menopausal women separately [19], 1 study of pre-menopausal [18], 4 studies of post-menopausal [15,33,35,36], and 5 studies of combined women [16,17,30,32,34]. Five studies were reported from Europe [18,20,32,34,35], 4 studies from USA [15,19,33,36], and 2 studies from Asia [16,17]. NOS stars of all included studies ranged from 4 to 10, with an average of 7.55. High quality studies (NOS stars ≥ 8) accounted for 55% of all studies [15-17,19,33,36], and moderate quality studies (6 ≤ NOS stars ≤ 7) accounted for 45% of all studies [18,20,32,34,35].

**Table 1:** Characteristics of included prospective studies

**Study (nation)**	**Design**	**Population (case/participants)**	**Menopausal status**	**Follow-up duration (years)**	**Exposure**		**Outcomes (RRs, 95% CI)**	**Study quality**
					**Measurement**	**Range (H vs. L)**^**a**^		
Vatten 1993 [18] (Norway)	NCC	Subjects from serum bank; 87/235;	Pre-	5	n-3/n-6: Serum PL, GC (mg/L)	0.36 vs. 0.14	1.0 (0.4, 2.1)	Moderate
								☆☆☆
								☆
								☆☆☆
Chajes 1999 [35] (Sweden)	NCC	Cardiovascular disease Cohort; 196/388;	Combined	2 ~ 11	LC n-3/n-6: Serum PL, GC (%tFC)	> 0.68 vs. <0.08	0.88 (0.42,1.86)	Moderate
								☆☆
								☆☆
								☆☆☆
Saadatian-Elahi 2002 [19] (USA)	NCC	Health university Women cohort; 197/197;	Pre- and Post-	4.3	n-3/n-6: Serum PL, GC (%tFC)	4th quantile vs. reference	Pre-: 0.60 (0.24, 1.54);	High ☆☆☆☆
								☆☆
								☆☆☆
							Post-: 0.42 (0.17, 1.08)	
Wirfalt 2002 [36] (Sweden)	NCC	Malmo Diet and Cancer (MDC) Cohort; 237/673;	Post-	3 ~ 8	n-3/n-6: Diet, FFQ (g/day)	0.33 vs. 0.15	0.66 (0.41, 1.08)	Moderate
								☆☆
								☆☆
								☆☆
Wakai 2005 [17] (Japan)	PC	Japan Collaborative Cohort Study (JACC);129/26291	Post- and Combined	7.6	n-6/n-3: Diet, FFQ (% energy)	Combined: > 4.61vs. < 3.25	Combined: 1.31 (0.78, 2.19)	High
								☆☆☆
								☆☆
								☆☆☆
						Post-: > 4.59 vs. < 3.21	Post-: 1.30 (0.66, 2.58)	
Chajes 2008 [20] (Sweden)	NCC	Europe Prospective Investigation into Cancer and Nutrition (EPIC); 363/702;	Combined.	7.0	n-6/n-3: Serum PL, GC (%tFC)	5th quantile vs. reference	0.76 (0.48, 1.20)	Moderate
								☆☆☆
								☆☆
								☆☆
Takata 2009 [37] (USA)	NCC	Beta Carotene and Retinol Efficacy Trial chort study (CARET); 103/309;	Post-.	4.4	n-3/n-6: Serum PL, GC (%tFC)	> 0.15 vs. < 0.11	0.74 (0.40, 1.36)	High
								☆☆☆
								☆☆
								☆☆☆
Thiebaut 2009 [33] (France)	PC	EPIC Cohort; 1650/56007	Combined	8.0	n-6/n-3:Diet, FFQ, (% energy)	14.76 vs. 5.48	0.97 (0.83, 1.14)	Moderate
								☆☆
								☆☆
								☆☆
Murff 2011 [16] (China)	PC	Shanghai Women Health Study cohort (SWHS); 712/72571;	Combined	8.0	n-6/n-3: Diet, FFQ, (g/day)	7.64 vs.5.18	1.02 (0.77, 1.34)	High
								☆☆☆☆
								☆☆
								☆☆☆
Park 2012 [15] (USA)	PC	Multiethnic Cohort; 3885/85089;	Post-	12	n-6/n-3: Diet, FFQ, (g/1000 kcal)	> 9.60 vs. < 7.60	1.10 (0.99, 1.22)	High
								☆☆☆☆
								☆☆
								☆☆
Sczaniecka 2012 [34] (USA)	PC	Vitamins and Lifestyle (VITAL) cohort study; 772/30252;	Post-.	6.0	n-3/n-6: Diet, FFQ, (g/day)	> 0.03 vs. < 0.005	0.84 (0.65, 1.09)	High
								☆☆☆
								☆☆
								☆☆☆

^a^H vs. L: the highest exposure quantile vs. lowest or reference.

%tFC = percentage of total Fatty Acid; GC = Gas Chromatography; LC n-3 = long chain n-3 PUFAs including EPA, DPA and DHA; NCC = prospective nested case–control study; PC = prospective cohort study; n-3/n-6 = ratio of n-3/n-6 polyunsaturated fatty acids; n-6/n-3 = ratio of n-6/n-3 polyunsaturated fatty acids; FFQ = food frequency questionnaire; Serum PL = serum phospholipids.

### Highest vs. lowest quantile of ratio of n-3/n-6 PUFAs

**Figure 2:**
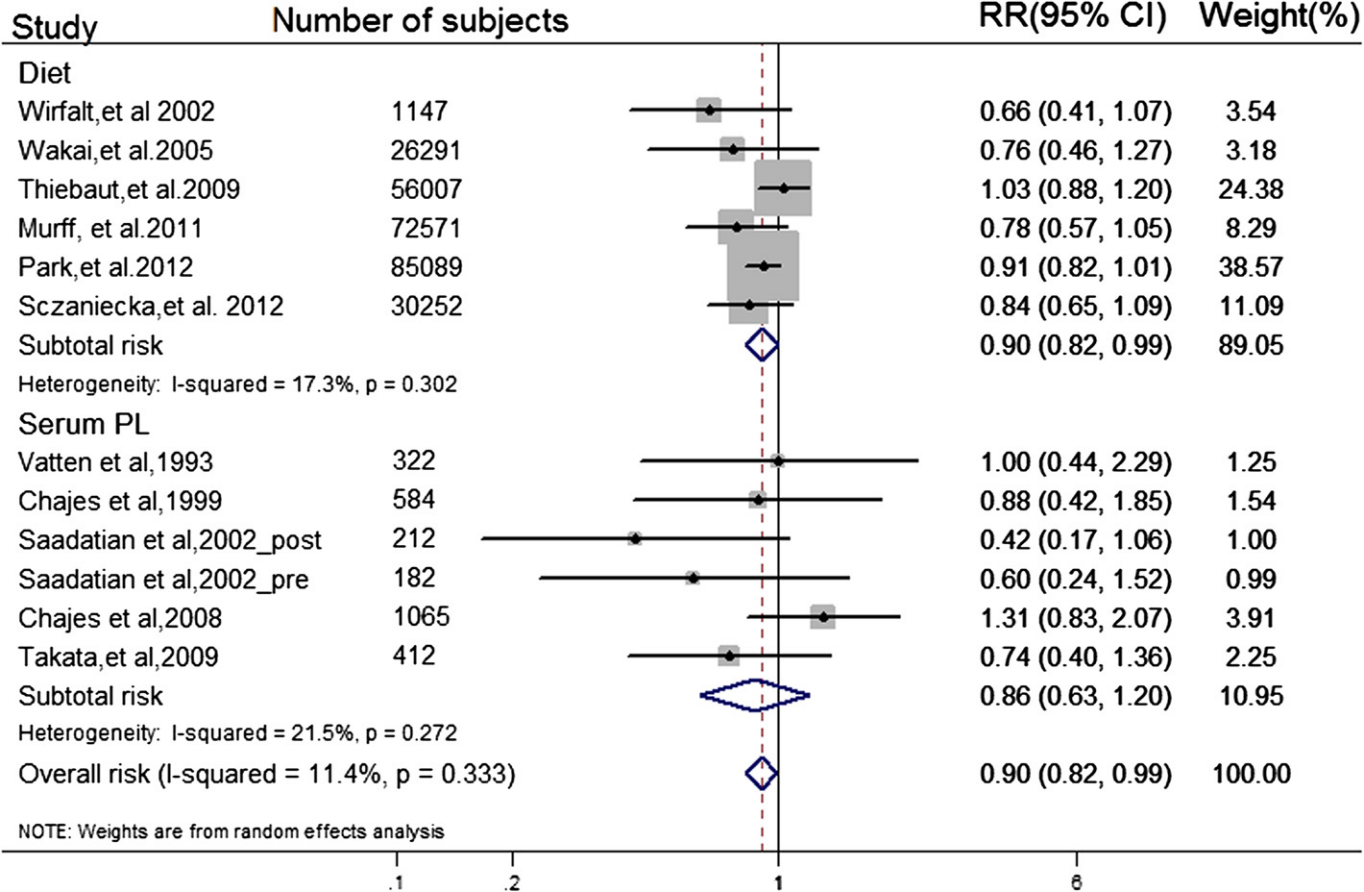
**Forest plot of ratio of n-3/n-6 PUFAs in diet or serum PL for the highest vs. lowest.** Prospective studies concerning dietary and serum PL ratio of n-3/n-6 PUFAs separately are referred to by first author, year of publication and number of subjects, weighted and ranked according to the inverse of the variance of the logRR estimate. The relative risks (RRs) are represented by the squares (the size is proportional to the weights used in the meta-analysis), and CIs are represented by the error bars. *P* values for heterogeneity test (I square and Q test) and RR for the highest exposure quantile vs. lowest from individual study were pooled by using random effect model. The diamonds can represent the pooled RR from subtotal risk estimate of dietary or serum PL ratio, according to their corresponding position in the figure.

We performed a random-effects model meta-analysis for highest quantile compared with lowest (Figure 2). Eleven independent prospective studies reported the association between ratio of n-3/n-6 and risk of breast cancer, involving 8,331 BC events and 274,135 adult females (premenopausal, postmenopausal, or combined) across different countries. Intake ratio of n-3/n-6 PUFA was inversely associated with BC risk for the highest vs. lowest quantile among study populations (pooled RR = 0.90; 95% CI: 0.82, 0.99; *I*^2^ = 11.40%; *P*_heterogeneity_ = 0.33).

### Summarized dose–response meta-analysis

**Figure 3:**
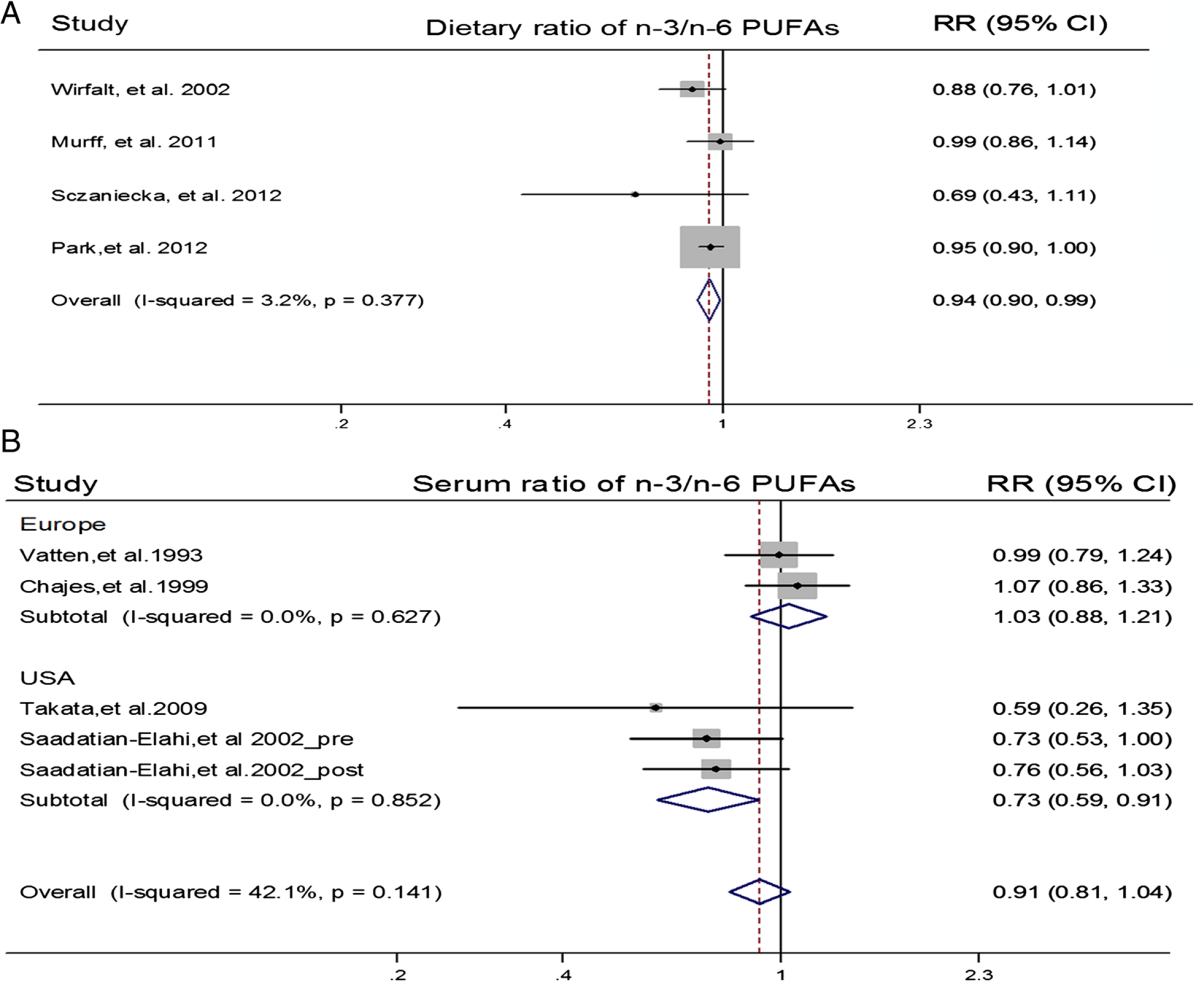
**Forest plot of per 1/10 increment of n-3/n-6 PUFAs ratio in diet and serum PL.** Prospective studies eligible for dose–response analysis are referred to by first author and year of publication. The relative risks (RRs) are represented by the squares (the size is proportional to the weights used in the meta-analysis), and confidence intervals (CIs) are represented by the error bars. *P* values and *I* square for heterogeneity test were shown by using random effect model. The diamonds can separately represent the pooled RR for association between per 1/10 increment of dietary or serum PL ratio of n-3/n-6 PUFA and BC risk, which was combined by a two-stage random-effect model. Figure **A** indicated association of BC risk with per 1/10 dietary ratio of n-3/n-6 PUFA, whereas Figure **B** indicated association of BC risk with pre 1/10 serum PL ratio of n-3/n-6 PUFA.

**Figure 4:**
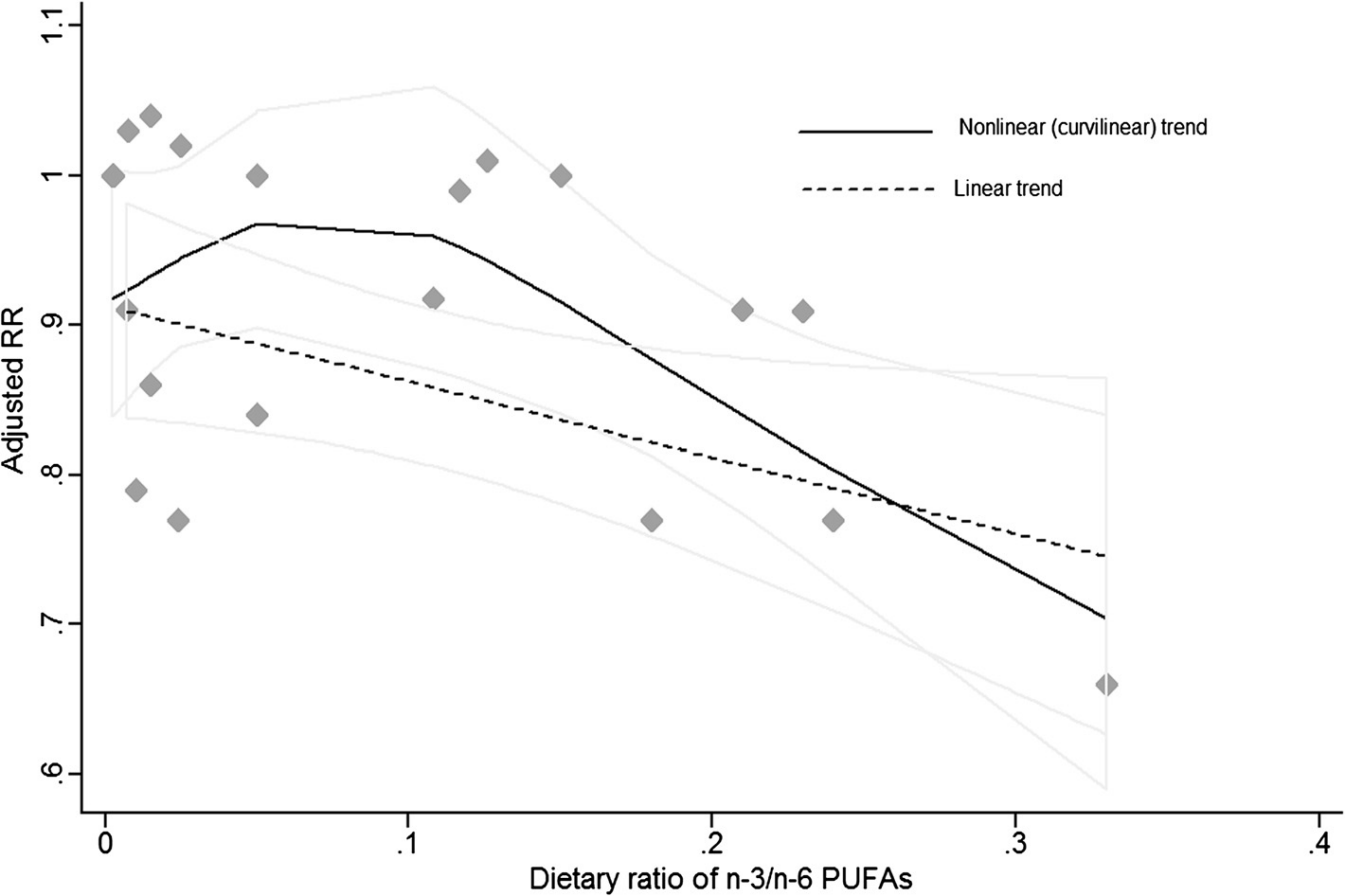
**Summarized dose–response association between dietary ratio of n-3/n-6 PUFAs and risk of breast cancer.** Adjusted RRs from each exposure quantile of dietary ratio of n-3/n-6 PUFAs in included individual studies were represented by the gray diamonds, and corresponding intervals (CIs) were represented by the lightgray trendline. The dash line indicated that dose–response linear trend (*P*_trend_ = 0.012) between dietary ratio of n-3/n-6 PUFAs and risk of breast cancer by use of variance-weighted least squares regression of fixed effect model; the black curve indicated nonlinear (curvilinear) trend (*P*_trend_ = 0.018) by use of restricted cubic splines functional model with three knots at percentiles 25%, 50%, and 75% of the distribution.

We performed summarized dose–response meta-analysis to determine the potential linear and curvilinear trend. Four articles reporting PUFA as g/day were eligible for the dose–response association between dietary ratio and BC risk [15,16,33,35]. There was statistical significance of dose–response trend (*P*_linear_ = 0.012; *P*_curvilinear_ = 0.018) among study populations. Per 1/10 increment of dietary n-3/n-6 ratio was associated with a 6% reduction of BC risk (pooled RR = 0.94; 95% CI: 0.90, 0.99; *I*^*2*^ = 3.20%, *P*_heterogeneity_ = 0.38) (Figures 3 &4).

Four articles reporting PUFAs as compositions were eligible for the dose–response association of serum PL biomarker with BC risk [15,16,18,19,33-36]. There was no statistical significance of dose–response trend (*P*_linear_ = 0.178; *P*_curvilinear_ = 0.832) among study populations, and the pooled RR for per 1/10 increment of n-3/n-6 ratio in serum PL was 0.89 (95% CI: 0.74, 1.06; *I*^*2*^ = 42.00%, *P*_heterogeneity_ = 0.14) (Figure 3). Further stratifying by geographical regions, there was statistical significance of dose–response trend among USA populations from 2 studies (*P*_linear_ = 0.004; *P*_curvilinear_ = 0.09), and per 1/10 increment of n-3/n-6 ratio in serum PL was associated with a 27% reduction of BC risk (pooled RR = 0.73; 95% CI: 0.59, 0.91; *I*^*2*^ = 0.00%). There was no statistical significance of dose–response trend among Europe populations from 2 studies (*P*_linear_ = 0.70; *P*_curvilinear_ = 0.95), and the pooled RR of per 1/10 increment of ratio in serum PL was 1.03 (95% CI: 0.88, 1.21; *I*^*2*^ = 0.00%) (Figure 3; Additional file 3). However, a permutation test from metaregression did not show significant difference between the two populations (*P* for metaregression = 0.086; *P* for a permutation test = 0.116).

### Subgroup analysis of ratio of n-3/n-6 PUFA for highest quantile vs. lowest

Metaregression and subgroup analysis was performed to explore the probable source of heterogeneity (Table 2). Although the association of increased ratio of n-3/n-6 with decreased risk of BC among 98862 participants from 2 Asian studies was more evident than that among 116,147 participants from 4 USA studies (pooled RR = 0.88; 95% CI: 0.80, 0.97; *I*^*2*^ = 0.00%) and 59,125 participants form 5 European studies (pooled RR = 1.00; 95% CI: 0.84, 1.18; *I*^*2*^ = 8.90%), results of metaregression did not show a significant difference between the three populations. When stratified by the adjustment for potential confounders, metaregression did not show a significant difference between estimates adjusted and those not adjusted for specific covariates.

**Table 2:** Subgroup analysis of intake n-3/n-6 PUFAs ratio for highest quantile vs. lowest

**Subgroup factors**	***N***	**Pooled RR (95% CI)**	**Heterogeneity**			***P***^***b***^	***P***^***c***^
			***I***^***2***^	**Degree**	***P***^***a***^		
Overall analysis	11	0.90 (0.82, 0.99)	11.40%	Low	0.33		
Study design						0.53	0.60
PC	5	0.92 (0.84, 1.00)	7.70%	Low	0.37		
NCC	6	0.82 (0.62, 1.08)	20.60%	Low	0.27		
Nations						0.07	0.12
Europe	5	1.00 (0.84, 1.18)	8.90%	Low	0.36		
USA	4	0.88 (0.80, 0.97)	0.00%	Low	0.95		
Asia	2	0.77 (0.59, 1.00)	0.00%	Low	0.42		
Menopausal status						0.18	0.26
Pre-	2	0.80 (0.43,1.48)	0.00%	Low	0.42		
Post-	5	0.85 (0.75.0.97)	0.00%	Low	0.33		
Combined	5	0.96 (0.80, 1.14)	23.30%	Low	0.27		
Exposure assessment						0.87	0.99
Serum PL biomarker	5	0.86 (0.63, 1.20)	21.50%	Low	0.27		
Diet	6	0.90 (0.82, 0.99)	17.30%	Low	0.30		
Follow-up duration						0.11	0.08
≤ Average value	6	0.77 (0.46, 0.94)	0.00%	Low	0.77		
> Average value	5	0.94 (0.83, 1.06)	19.50%	Low	0.21		
Covariates adjusted							
BMI						0.24	0.36
Yes	8	0.91 (0.83, 1.00)	9.90%	Low	0.35		
No	3	0.65 (0.39,1.09)	0.00%	Low	0.38		
Age of fist childbirth						0.09	0.07
Yes	7	0.87 (0.79, 0.94)	0.00%	Low	0.56		
No	4	1.01 (0.70, 1.12)	0.00%	Low	0.54		
Age at menarche						0.51	0.54
Yes	6	0.92 (0.85, 0.99)	0.00%	Low	0.51		
No	5	0.80 (0.58,1.11)	33.60%	Moderate	0.18		
Parity						0.11	0.08
Yes	5	0.94 (0.85, 1.04)	15.90%	Low	0.31		
No	6	0.77 (0.63, 0.94)	0.00%	Low	0.68		
Reproductive variables						0.65	0.76
Yes	4	0.89 (0.81, 0.98)	0.00%	Low	0.73		
No	7	0.89 (0.74, 1.07)	33.70%	Moderate	0.16		
Family history of BC						0.30	0.30
Yes	8	0.91 (0.81, 1.02)	31.30%	Moderate	0.18		
No	3	0.76 (0.56,1.04)	0.00%	Low	0.83		
Hormone user						0.75	0.90
Yes	9	0.89 (0.80, 1.00)	24.50%	Low	0.22		
No	2	0.82 (0.50, 1.35)	0.00%	Low	0.57		
Alcohol intake						0.32	0.32
Yes	9	0.91 (0.82, 1.00)	0.00%	Low	0.26		
No	2	0.72 (0.47, 1.10)	20.60%	Low	0.51		
Smoking						0.23	0.28
Yes	4	0.94 (0.86, 1.02)	0.00%	Low	0.40		
No	7	0.83 (0.70, 0.98)	8.20%	Low	0.37		
Age						0.78	0.99
Yes	6	0.92 (0.82, 1.02)	0.00%	Low	0.45		
No	5	0.86 (0.68, 1.10)	34.00%	Moderate	0.18		
Educational status						0.16	0.14
Yes	5	0.87 (0.80, 0.96)	0.00%	Low	0.59		
No	6	0.96 (0.79, 1.17)	14.70%	Low	0.32		
Total energy intake						0.16	0.16
Yes	5	0.87 (0.80, 0.96)	0.00%	Low	0.59		
No	6	0.96 (0.79, 1.17)	14.70%	Low	0.32		
Physical activity						0.20	0.20
Yes	3	0.81 (0.67, 0.97)	0.00%	Low	0.90		
No	8	0.92 (0.81, 1.05)	21.50%	Low	0.25		
Other drugs or nutrients intake						0.20	0.24
Yes	3	0.81 (0.67, 0.97)	0.00%	Low	0.90		
No	8	0.92 (0.81, 1.05)	21.50%	Low	0.25		

^*a*^*P*: *P* value for heterogeneity within subgroup.

^*b*^*P*: *P* value for heterogeneity between subgroups with a meta-regression analysis.

^*c*^*P*: adjusted *P* value for heterogeneity between subgroups with a permutation test.

*N* = number of studies.

When separately analyzed by exposure assessment, we found a significantly negative association between BC risk and n-3/n-6 ratio in diet from 5 prospective cohort and 1 nested case–control studies, involving 7,385 BC invents and 271,357 participants (pooled RR = 0.92; 95% CI: 0.84, 1.00), whereas no significant association was found between BC risk and serum PL biomarker from 5 prospective nested case–control studies, involving 946 BC cases and 1832 controls (pooled RR = 0.86; 95% CI: 0.63, 1.20). However, there was no apparent difference between studies using diet ratio of n-3/n-6 as exposure and those using serum biomarker of n-3/n-6 as exposure with metaregression (*P* for metaregression = 0.870; *P* for a permutation test = 0.990).

### Sensitivity analysis and publication bias

Finally, we conducted sensitivity analysis and publication bias analysis (Table 3). For dietary n-3/n-6 PUFAs ratio as exposure, sensitivity analysis indicated that exclusion of any individual study did not substantially change the end results. However, for serum PL biomarker, the sensitivity analysis after sequentially omitting 1 study at a time and reanalyzing the remaining data showed that there was significantly negative association between serum PL n-3/n-6 ratio and BC risk (pooled RR = 0.73; 95% CI: 0.51, 1.01; *I*^*2*^ = 0.00%) after excluding the study by Chajes [20], indicating the overall risk estimation was substantially influenced by the single study [20].

**Table 3:** Sensitivity analysis for the relationship between intake n-3/n-6 PUFAs ratio and breast cancer risk

**Sensitivity analysis**	**Diet ratio of n-3/n-6**				**Serum PL ratio of n-3/n-6**				**Intake ratio of n-3/n-6**			
	**N**	**Pooled RR (95% CI)**	**Heterogeneity**		**N**	**Pooled RR(95% CI)**	**Heterogeneity**		**N**	**Pooled RR(95% CI)**	**Heterogeneity**	
			***I***^***2 ***^**Degree**	***P***			***I***^***2 ***^**Degree**	***P***			***I***^***2 ***^**Degree**	***P***
Exclusion of studies with potential selection bias [20,32]	5	0.87 (0.80, 0.96)	0.00% (Low)	0.5s9	4	0.73 (0.51, 1.01)	0.00% (Low)	0.67	9	0.86 (0.79, 0.94)	0.00% (Low)	0.72
Exclusion of studies without covariates adjusted [18]	6	0.90 (0.82, 0.99)	17.30% (Low)	0.30	4	0.82 (0.56, 1.22)	36.50% (Low)	0.17	10	0.89 (0.81, 0.99)	19.10% (Low)	0.26
Contour-enhanced funnel plots of fixed effect model	6	0.91 (0.84, 0.98)	17.30% (Low)	0.30	5	0.90 (0.68, 1.19)	21.50% (Low)	0.27	11	0.91 (0.84, 0.98)	11.40% (Low)	0.33
Contour-enhanced funnel plots of random effect model	6	0.90 (0.82, 0.99)	17.30% (Low)	0.30	5	0.87 (0.63, 1.20)	21.50% (Low)	0.27	11	0.90 (0.82, 0.98)	11.40% (Low)	0.33

*N =* number of studies; *P* = *P* value for heterogeneity within subgroup.

In publication bias analysis, visual inspection of Begg’s funnel plot (*P* = 0.244) and Egger’s regression test (*P* = 0.138) showed no evidence of possible publication bias. Contour-enhanced funnel plots of random effect model showed studies appear to be missing in areas of high statistical significance (p < 0.05), indicating that publication bias is a less likely cause of the funnel asymmetry (Additional file 3).

## Discussion

The present study, involving 8,331 BC events from 274,135 participants, indicated that higher ratio of n-3/n-6 PUFAs is associated with lower risk of BC, and per 1/10 increment of n-3/n-6 ratio in diet is associated with a 6% reduction of BC risk. However, subgroup analysis showed there was no significant relationship between serum PL biomarker and BC risk, but the significant difference between association of dietary n-3/n-6 ratio and serum PL with BC risk was not observed with metaregression (*P* for metaregression = 0.87; *P* for a permutation test = 0.99). Fatty acid profile in serum phospholipids may be an untypical representative of sensitive biomarkers indicating post-absorptive amounts and change at the target tissue, which did not provide the same information as the dietary questionnaire tools. Therefore, the strength of the relationship between the ratio in serum PL and BC risk might be lower compared with the relationship between the ratio in diet and the risk of BC.

Although the was no significant difference between most subgroups with metaregression, study heterogeneity (*I*^*2*^ = 21.00%) in this meta-analysis indicated the subtotal variation was attributable to between individual studies. There are some possible reasons for explaining the potential heterogeneity in present study. Firstly, the heterogeneity from geographic regions could be partially explained by different dietary patterns. The n-3/n-6 ratio in Japanese diet is about 1 to 4, whereas that in western diet (USA and Europe) is about 1 to 15–20 [37]. Imbalance of n-3/n-6 PUFAs ratio in diet across different countries is the consequence of excessive n-6 PUFAs consumption largely from corn, sunflower or safflower oils in the Western diet, but higher n-3 PUFAs intake largely from marine foods in the Asian diet, particularly in the Japanese population, which partially explains the inconsistency between Asia and Western studies in this meta-analysis. Subsequently, lower n-3/n-6 ratio in the diet will lead to lower ratio of n-3/n-6 PUFAs in serum PL. One randomized clinical trial showed that plasma n-3/n-6 ratio significantly increased among subjects after lovaza intervention (a prescription-strength pill, 4 g/day, EPA + DHA = 3.36 g, duration for 2 years) compared with subjects without intervention [38]. *In vivo*, AA (20:4n-6, arachidonic acid) and EPA (20:5n-3, eicosapentaenoic acid) can simultaneously compete for the same cyclooxygenases (COX) and lipoxygenases (LOX) metabolism pathways, leading to the production of n-6 family derived 2-series PG and 4-series LT with promoting tumor growth effects [39,40], and n-3 family derived 3-series PG and 5-series LT with suppressive effects [41,42]. Thus, the higher ratio of n-3/n-6 PUFA entering the cellular pool from dietary sources could be involved in BC carcinogenesis by changing the balance of tissue eicosanoids. Finally, there is evidence that change of estrogen metabolism is probably involved in mammary carcinogenesis among post-menopausal females [43]. EPA/AA ratio present in cell membrane lipids could influence the balance of prostaglandin E_**3**_ (PGE_3_)/prostaglandin E_2_ (PGE_2_) to inactivate the activity of adipose aromatase P 450 which catalyzes the conversion of 19-carbon steroids to estrogens, and thus reduce estrogen-stimulated cell growth action [44,45].

This meta-analysis had several strengths. Firstly, the quantitative assessment was based on data from prospective cohort studies. This minimizes the possibility that overall analysis will be influenced due to recall bias, which could be of more common concern in retrospective case–control studies. Also, we had higher statistical power to estimate the relationship between intake ratio of n-3/n-6 PUFA and risk of BC by analyzing summary data from 274,135 participants in 11 prospective studies. Subsequently, serum PL as a biomarker directly reflecting intake ratio of n-3/n-6 fatty acids has the advantage of providing more objective ratio of n-3/n-6 PUFA *in vivo*, independent of subjective recall bias and probable measurable errors owing to dietary questionnaire tools used. Finally, more than 50% of the studies were high quality studies (NOS stars ≥ 8), and exclusion of any individual study did not significantly change the negative relationship between intake ratio of n-3/n-6 PUFAs and BC risk. Although publication bias could be of concern because small studies with null results tend not to be published, there was no evidence of potential publication bias in the present meta-analysis.

Meta-analyses of observational studies are susceptible to methodological and confounding biases inherent in the original studies. Therefore, there are also several limitations considered in our study. Firstly, although prospective studies are more superior to retrospective case–control studies with regards to elucidating causal relationships, selection bias from study populations might be still unavoidable. Volunteers recruited from occupational exposure populations (e.g., teacher, workers and nurse) might not be an unbiased representative of study population. Subsequently, although the fatty acid profile in serum PL, implying absorption and circulation transportation mechanisms, reflects acute dietary intakes over the past few days, it may be a untypical representation of sensitive biomarkers indicating post-absorptive amounts and change at the target tissue. Moreover, any measurement error and resulting misclassification would most likely lead to an attenuation of the true association. Intake of fatty acids was evaluated using dietary questionnaire tools, which could potentially have measurable errors, due to inaccurate fatty acids database and diet reporting bias. Similarly, fatty acids in serum PL,, especially long chain PUFAs, are probably undergoing change during the long follow-up time and storage time [46], due to double bonds being easily oxidized. Consequently, potential measurement bias might lead to underestimation or overestimation of risk of BC [47]. Although only original studies with adjusting for at least three covariates were included in the present study, the possibility of altering summary results from residual confounding factors still cannot be excluded. Data extraction and analyses were not blinded to the authors and publication agencies, and the references screening and data extraction were conducted independently and in duplicate by 2 investigators, therefore data selection bias was unlikely. Although reporting bias might be present in our study, due to English language bias from exclusion of non-English language articles, our eligible studies covered a wide range of non-English countries, such as countries across Europe and Asia, which partially eliminated the possible effects of report bias on overall risk estimates. Finally, the limited numbers of studies included in each subgroup might diminish the statistical power to detect the association between ratio of n-3/n-6 PUFA and BC risk. Although we separately made a stratified analysis for association of serum PL ratio with BC risk and dose–response trend, it is difficult to find firm evidences among study populations within subgroup analysis, particular among USA females (*P* for metaregression = 0.103; *P* for a permutation test = 0.100).

The present meta-analysis has also important medical implications of balanced n-3/n-6 fatty acid intake ratio for BC prevention, clinical diagnosis and treatment. Firstly, although many sensitive tumor biomarkers were used for the clinical diagnosis of BC, several potential limitations were still unavoidable. In addition, the BC inflammatory microenvironment plays a major role in growth, invasiveness and resistance to therapy. However, modulation ratio of n-3/n-6 fatty acids in food, as a potent inducer of metabolic responses, could provide an effective means to alter ratio of n-3/n-6 fatty acid in tumor tissue and thereby possibly affect tumor growth, by sensitizing cancer cells to chemotherapy and increasing resistance of normal cells to the toxic effects. Finally, diet intervention could represent a clinically relevant adjuvant therapy in patients with BC, based on the general tenet of clinical nutrition.

## Conclusions

In summary, findings from this meta-analysis of prospective studies provide a conclusive evidence to support increase intake ratio of n-3/n-6 PUFAs for BC prevention among study females. Quantitative conclusions from the present study showed that per 1/10 increment of n-3/n-6 ratio in diet, there was a 6% reduction of BC risk among study populations (USA, Europe and Asia). This important evidence highlights the need to promote nutritional education programs, stressing the need to increase the consumption of food rich in n-3 PUFAs (marine foods), decrease the consumption of food rich in n-6 PUFAs (vegetable oils and processed foods), to ultimately improve intake ratio of n-3/n-6 PUFAs. However, no conclusive evidence from USA populations can be obtained and this needs to be,further confirmed by prospective population-based study on tissue biomarker of n-3/n-6 PUFAs ratio, and larger randomized controlled trials are required to determine whether higher intake ratio of n-3/n-6 PUFAs will have beneficial effects on BC risk, or improve the prognosis of patients with BC.

## Competing interests

The authors declare that they have no competing interests.

## Authors’ contributions

BY and XR had full access to all of the data in the study, and take responsibility for the integrity of the data and the accuracy of the data analysis. BY XR and DL participated in study conception and design. YF and JG carried out literature research, study identification, data extraction and organization. BY and RX performed study quality assessment and statistical analysis. BY finished drafting of the manuscript; DL XR and BY carried out critical revision of the manuscript. All authors read and approved the final manuscript submitted for publication and are guarantors for the study.

## Pre-publication history

The pre-publication history for this paper can be accessed here:

http://www.biomedcentral.com/1471-2407/14/105/prepub

## Supplementary Material

Additional file 1 **Completed reporting items for systematic review and meta-Analysis (MOOSE Checklist) of present study.** Information as to how this article was conducted. Click here for file

Additional file 2 **Completed data extraction from eligible original studies.** Information as to how the available data were extracted by using the standard extraction forms. Click here for file

Additional file 3: Table S1 Details on data synthesis and analysis in this meta-analysis. Studies excluded after scrutiny with reasons for exclusion. **Table S2.** Study quality assessment of included prospective studies by Newcastle-Ottawa Scale. **Figure S1.** Dose–response trend between serum PL ratio of n-3/n-6 PUFAs and breast cancer risk among USA females. **Figure S2.** Contour-enhanced funnel plots for association of ratio of n-3/n-6 PUFA with BC risk. Click here for file

## References

[B1] Parkin DM Bray F Ferlay J Pisani P Estimating the world cancer burden: Globocan 2000 Int J Cancer 2001 94 2 153 156 10.1002/ijc.1440 11668491

[B2] Bhoo-Pathy N Yip CH Hartman M Uiterwaal CS Devi BC Peeters PH Taib NA van Gils CH Verkooijen HM Breast cancer research in Asia: adopt or adapt Western knowledge? Eur J Cancer 2013 49 3 703 709 10.1016/j.ejca.2012.09.014 23040889

[B3] Shimizu H Ross RK Bernstein L Yatani R Henderson BE Mack TM Cancers of the prostate and breast among Japanese and white immigrants in Los Angeles County Br J Cancer 1991 63 6 963 966 10.1038/bjc.1991.210 2069852 PMC1972548

[B4] Kim EH Willett WC Colditz GA Hankinson SE Stampfer MJ Hunter DJ Rosner B Holmes MD Dietary fat and risk of postmenopausal breast cancer in a 20-year follow-up Am J Epidemiol 2006 164 10 990 997 10.1093/aje/kwj309 16968865

[B5] Zhang CX Ho SC Lin FY Chen YM Cheng SZ Fu JH Dietary fat intake and risk of breast cancer: A case–control study in China Eur J Cancer Prev 2011 20 3 199 206 10.1097/CEJ.0b013e32834572bb 21403522

[B6] MacLennan M Ma DWL Role of dietary fatty acids in mammary gland development and breast cancer Breast Cancer Res 2010 12 5 211 221 10.1186/bcr2646 21067551 PMC3096965

[B7] Karmali RA Eicosanoids in neoplasia Prev Med 1987 16 4 493 502 10.1016/0091-7435(87)90063-6 3114735

[B8] Wen ZH Su YC Lai PL Zhang Y Xu YF Zhao A Yao GY Jia CH Lin J Xu S Wang L Wang XK Liu AL Jiang Y Dai YF Bai XC Critical role of arachidonic acid-activated mTOR signaling in breast carcinogenesis and angiogenesis Oncogene 2013 32 2 160 170 10.1038/onc.2012.47 22349822

[B9] Vendramini-Costa DB Carvalho JE Molecular link mechanisms between inflammation and cancer Curr Pharm Des 2012 18 26 3831 3852 10.2174/138161212802083707 22632748

[B10] Chamras H Ardashian A Heber D Glaspy JA Fatty acid modulation of MCF-7 human breast cancer cell proliferation, apoptosis and differentiation J Nutr Biochem 2002 13 12 711 716 10.1016/S0955-2863(02)00230-9 12550055

[B11] Stillwell W Shaikh SR Zerouga M Siddiqui R Wassall SR Docosahexaenoic acid affects cell signaling by altering lipid rafts Reprod Nutr Dev 2005 45 5 559 579 10.1051/rnd:2005046 16188208

[B12] Shannon J King IB Lampe JW Gao DL Ray RM Lin MG Stalsberg H Thomas DB Erythrocyte fatty acids and risk of proliferative and nonproliferative fibrocystic disease in women in Shanghai China. Am J Clin Nutr 2009 89 1 265 276 10.3945/ajcn.2008.26077 PMC2647713 19056601

[B13] Kuriki K Hirose K Wakai K Matsuo K Ito H Suzuki T Hiraki A Saito T Iwata H Tatematsu M Tajima K Breast cancer risk and erythrocyte compositions of n-3 highly unsaturated fatty acids in Japanese Int J Cancer 2007 121 2 377 385 10.1002/ijc.22682 17354239

[B14] Maillard V Bougnoux P Ferrari P Jourdan ML Pinault M Lavillonniere F Body G Le Floch O Chajes V N-3 and N-6 fatty acids in breast adipose tissue and relative risk of breast cancer in a case–control study in Tours France. Int J Cancer 2002 98 1 78 83 10.1002/ijc.10130 11857389

[B15] Park SY Kolonel LN Henderson BE Wilkens LR Dietary fat and breast cancer in postmenopausal women according to ethnicity and hormone receptor status: the multiethnic cohort study Cancer Prev Res (Phila) 2012 5 2 216 228 10.1158/1940-6207.CAPR-11-0260 22166249 PMC4495954

[B16] Murff HJ Shu XO Li HL Yang G Wu XY Cai H Wen WQ Gao YT Zheng W Dietary polyunsaturated fatty acids and breast cancer risk in Chinese women: a prospective cohort study Int J Cancer 2011 128 6 1434 1441 10.1002/ijc.25703 20878979 PMC3086389

[B17] Wakai K Tamakoshi K Date C Fukui M Suzuki S Lin Y Niwa Y Nishio K Yatsuya H Kondo T Tokudome S Yamamoto A Toyoshima H Tamakoshi A JACC Study Group Dietary intakes of fat and fatty acids and risk of breast cancer: A prospective study in Japan Cancer Sci 2005 96 9 590 599 10.1111/j.1349-7006.2005.00084.x 16128744 PMC11159093

[B18] Vatten LJ Bjerve KS Andersen A Jellum E Polyunsaturated fatty acids in serum phospholipids and risk of breast cancer: a case–control study from the Janus serum bank in Norway Eur J Cancer 1993 29A 4 532 538 8435206 10.1016/s0959-8049(05)80146-7

[B19] Saadatian-Elahi M Toniolo P Ferrari P Goudable J Akhmedkhanov A Zeleniuch-Jacquotte A Riboli E Serum fatty acids and risk of breast cancer in a nested case–control study of the New York University Women’s Health Study Cancer Epidem Biomar 2002 11 11 1353 1360 12433711

[B20] Chajes V Thiebaut ACM Rotival M Gauthier E Maillard V Boutron-Ruault MC Joulin V Lenoir GM Clavel-Chapelon F Association between serum trans-monounsaturated fatty acids and breast cancer risk in the E3N-EPIC study Am J Epidemiol 2008 167 11 1312 1320 10.1093/aje/kwn069 18390841 PMC2679982

[B21] Stroup DF Berlin JA Morton SC Olkin I Williamson GD Rennie D Moher D Becker BJ Sipe TA Thacker SB Meta-analysis of observational studies in epidemiology: a proposal for reporting. Meta-analysis Of Observational Studies in Epidemiology (MOOSE) group JAMA 2000 283 15 2008 2012 10.1001/jama.283.15.2008 10789670

[B22] Wells GA SB O'Connell D Peterson J Welch V Losos M The Newcastle-Ottawa Scale (NOS) for assessing the quality of non randomized studies in meta-analyses 2011 http://www.ohri.ca/programs/clinical_epidemiology/oxford.htm. Accessed Novenber 21, 2012

[B23] DerSimonian R Laird N Meta-analysis in clinical trials Control Clin Trials 1986 7 3 177 188 10.1016/0197-2456(86)90046-2 3802833

[B24] Greenland S Longnecker MP Methods for trend estimation from summarized dose–response data, with applications to meta-analysis Am J Epidemiol 1992 135 11 1301 1309 1626547 10.1093/oxfordjournals.aje.a116237

[B25] Orsini N Bellocco R Greenland S Generalized least squares for trend estimation of summarized dose–response data Stata J 2006 6 1 40 57

[B26] Larsson SC Orsini N Wolk A Vitamin B6 and risk of colorectal cancer: a meta-analysis of prospective studies JAMA 2010 303 11 1077 1083 10.1001/jama.2010.263 20233826

[B27] Harrell FE Jr Lee KL Pollock BG Regression models in clinical studies: determining relationships between predictors and response J Natl Cancer Inst 1988 80 15 1198 1202 10.1093/jnci/80.15.1198 3047407

[B28] Higgins JP Thompson SG Deeks JJ Altman DG Measuring inconsistency in meta-analyses BMJ 2003 327 7414 557 560 10.1136/bmj.327.7414.557 12958120 PMC192859

[B29] Egger M Davey Smith G Schneider M Minder C Bias in meta-analysis detected by a simple, graphical test BMJ 1997 315 7109 629 634 10.1136/bmj.315.7109.629 9310563 PMC2127453

[B30] Peters JL Sutton AJ Jones DR Abrams KR Rushton L Contour-enhanced meta-analysis funnel plots help distinguish publication bias from other causes of asymmetry J Clin Epidemiol 2008 61 10 991 996 10.1016/j.jclinepi.2007.11.010 18538991

[B31] Duval S Tweedie R Trim and fill: a simple funnel-plot-based method of testing and adjusting for publication bias in meta-analysis Biometrics 2000 56 2 455 463 10.1111/j.0006-341X.2000.00455.x 10877304

[B32] Thiebaut AC Chajes V Gerber M Boutron-Ruault MC Joulin V Lenoir G Berrino F Riboli E Benichou J Clavel-Chapelon F Dietary intakes of omega-6 and omega-3 polyunsaturated fatty acids and the risk of breast cancer Int J Cancer 2009 124 924 931 2008/11/28 edn 10.1002/ijc.23980 19035453

[B33] Sczaniecka AK Brasky TM Lampe JW Patterson RE White E Dietary intake of specific fatty acids and breast cancer risk among postmenopausal women in the VITAL cohort Nutrition and cancer 2012 64 8 1131 1142 10.1080/01635581.2012.718033 23137008 PMC3633593

[B34] Chajes V Hulten K Van Kappel AL Winkvist A Kaaks R Hallmans G Lenner PG Riboli E Fatty-acid composition in serum phospholipids and risk of breast cancer: an incident case–control study in Sweden Int J Cancer 1999 83 5 585 590 10.1002/(SICI)1097-0215(19991126)83:5<585::AID-IJC2>3.0.CO;2-Z 10521790

[B35] Wirfalt E Mattisson I Gullberg B Johansson U Olsson H Berglund G Postmenopausal breast cancer is associated with high intakes of (omega)6 fatty acids (Sweden) Cancer Causes Control 2002 13 10 883 893 10.1023/A:1021922917489 12588084

[B36] Takata Y King IB Neuhouser ML Schaffer S Barnett M Thornquist M Peters U Goodman GE Association of serum phospholipid fatty acids with breast cancer risk among postmenopausal cigarette smokers Cancer Cause Control 2009 20 4 497 504 10.1007/s10552-009-9314-2 PMC2795699 19255861

[B37] Simopoulos AP The Mediterranean diets: What is so special about the diet of Greece? The scientific evidence J Nutr 2001 131 11 SUPPL 3065S 3073S 11694649 10.1093/jn/131.11.3065S

[B38] Signori C DuBrock C Richie JP Prokopczyk B Demers LM Hamilton C Hartman TJ Liao J El-Bayoumy K Manni A Administration of omega-3 fatty acids and Raloxifene to women at high risk of breast cancer: interim feasibility and biomarkers analysis from a clinical trial Eur J Clin Nutr 2012 66 8 878 884 10.1038/ejcn.2012.60 22669332

[B39] Reader J Holt D Fulton A Prostaglandin E2 EP receptors as therapeutic targets in breast cancer Cancer Metastasis Rev 2011 30 3–4 449 463 22002714 10.1007/s10555-011-9303-2 PMC3640271

[B40] Hopkins GJ Kennedy TG Carroll KK Polyunsaturated fatty acids as promoters of mammary carcinogenesis induced in Sprague–Dawley rats by 7,12-dimethylbenz[a]anthracene J Natl Cancer Inst 1981 66 3 517 522 6782319

[B41] Grammatikos SI Subbaiah PV Victor TA Miller WM n-3 and n-6 fatty acid processing and growth effects in neoplastic and non-cancerous human mammary epithelial cell lines Br J Cancer 1994 70 2 219 227 10.1038/bjc.1994.283 8054269 PMC2033515

[B42] Rovito D Giordano C Vizza D Plastina P Barone I Casaburi I Lanzino M De Amicis F Sisci D Mauro L Aquila S Catalano S Bonofiglio D Andò S Omega-3 PUFA ethanolamides DHEA and EPEA induce autophagy through PPAR(gamma) activation in MCF-7 breast cancer cells J Cell Physiol 2013 228 6 1314 1322 10.1002/jcp.24288 23168911

[B43] Rose DP Connolly JM Omega-3 fatty acids as cancer chemopreventive agents Pharmacol Ther 1999 83 3 217 244 10.1016/S0163-7258(99)00026-1 10576293

[B44] Obata T Nagakura T Masaki T Maekawa K Yamashita K Eicosapentaenoic acid inhibits prostaglandin D2 generation by inhibiting cyclo-oxygenase-2 in cultured human mast cells Clin Exp Allergy 1999 29 8 1129 1135 10.1046/j.1365-2222.1999.00604.x 10457118

[B45] Hamid R Singh J Reddy BS Cohen LA Inhibition by dietary menhaden oil of cyclooxygenase-1 and 2 in N-nitrosomethylurea-induced rat mammary tumors Int J Oncol 1999 14 3 523 528 10024686 10.3892/ijo.14.3.523

[B46] Pietinen P Vartiainen E Seppanen R Aro A Puska P Changes in diet in Finland from 1972 to 1992: Impact on coronary heart disease risk Prev Med 1996 25 3 243 250 10.1006/pmed.1996.0053 8781001

[B47] Nettleton JA Omega-3-fatty-acids - comparison of plant and seafood sources in human-nutrition J Am Diet Assoc 1991 91 3 331 337 1825498

