# Supplementary material for: Ratio of n-3/n-6 PUFAs and risk of breast cancer: a meta-analysis of 274135 adult females from 11 independent prospective studies
Source: BMC Cancer. 2014 Feb 18;14:105. doi: 10.1186/1471-2407-14-105 (PMC4016587; doi:10.1186/1471-2407-14-105)
Supplement: Additional file 1 — Completed reporting items for systematic review and meta-Analysis (MOOSE Checklist) of present study. Information as to how this article was conducted. [file 1471-2407-14-105-S1.pdf]

Additional file 1. MOOSE Checklist of Present Meta-Analysis

Criteria	Comments of how the criteria were handled in the meta-analysis	Reported on page #
	**Reporting of background should include**	
Problem definition	Breast cancer (BC) is one of the major public health problems among women worldwide, particularly in North America and Western Europe. Dietary PUFAs as a potentially dietary factor is closely correlated with increased BC incidence. Findings from prospective studies on ratio of n-3/n-6 PUFAs related to BC risk are still controversial; therefore, the potential public health impact of tissue or dietary ratio of n-3/n-6 PUFAs remains to be summarized quantitatively.	3
Hypothesis statement	Ratio of n-3/n-6 PUFAs from background diet and tissue probably plays an important role on the risk of human BC.	3
Description of study outcomes	However, there are some inconsistent conclusions in prospective studies, and the optimal dietary or tissue ratio of n-3/n-6 PUFAs in relation to BC has not yet been well defined.	4
Type of exposure or intervention used	Dietary or tissue ratio of n-3/n-6 PUFAs	4
Type of study designs used	Systematic review and meta-analysis.	4
Study population	Any aged adult females across different countries	4
	**Reporting of search strategy should include**	
Qualifications of searchers(eg. librarians and investigators)	Two trained reviewers (YF and JG) are indicated in the author list. Discrepancies unsolved by discussion during the course of study identification consulted to a third reviewer (BY).	5
Search strategy, including time period included in the synthesis and keywords	Search strategy was (*"Fatty Acids, Omega-3"[Mesh]OR "Fatty Acids, Omega-6"[Mesh]) AND "Breast Neoplasms"[Mesh]* for PubMed, *"Breast tumor" AND* (*"omega 3 fatty acid" OR "omega 6 fatty acid")* for EMBASE and *"Fatty Acids"[Mesh] AND "Breast Neoplasms"[Mesh]* for Cochrane Library databases.	4

Databases and registries searched	PubMed, EMBASE and Cochrane Library database were searched, and we also check the reference lists to identify studies that might have been missed..	4
Search software used, name and version, including special features	We did not employ search software. EndNote was used to merge retrieved citations and eliminate duplications	5, 6
Use of hand searching	We hand-searched bibliographies of retrieved papers, and check the reference lists from systematic review to identify studies that might have been missed.	4
List of citations located and those excluded, including justifications	The all steps and details of the literature search process are outlined in the flow chart (Figure 1; Additional file 4).	5
Method of addressing articles published in languages other than English	Our search was restricted to human studies, and studies published in English.	5, 6
Method of handling abstracts and unpublished studies	Abstract, unpublished studies and duplicated study were excluded	5
Description of any contact with authors	We did not contact authors for the detailed information of primary studies and unpublished studies.	6
	**Reporting of methods should include**	
Description of relevance or appropriateness of studies assembled for assessing the hypothesis to be tested	Detailed inclusion and exclusion criteria were described in the methods section.	5, 6
Rationale for the selection and coding of data	Data extracted from each of the studies were relevant to the population characteristics, study design, exposure, outcome, and adjusted confounding factors as covariates.	5, 6

Assessment of confounding	Restricted the analysis to multiple covariates adjusted estimates. Conducted sensitivity analyses by eliminating studies with possible selection bias. Publication bias was quantitatively examined by Begg's test and Egger's regression test. Contour-enhanced meta-analysis funnel plots was performed to differentiate asymmetry due to publication bias from that due to other factors, and provide a summary effect estimate before and after trim-fill algorithm based on all studies including the estimated missing studies.	7
Assessment of study quality, including blinding of quality assessors; stratification or regression on possible predictors of study results	We valuated study quality and risk of bias by using the Newcastle-Ottawa scale. Subgroup analyses were conducted to identify the sources of heterogeneity by study design, different regions, menopausal status, tissue types, study quality, and follow-up duration in included studies.	6, 7
Assessment of heterogeneity	Heterogeneity of the studies were explored within two types of study designs using Cochrane's Q test of heterogeneity and I^2 statistic that provides the relative amount of variance of the summary effect due to the between-study heterogeneity.	7
Description of statistical methods in sufficient detail to be replicated	Description of methods of meta-analyses for highest exposure quantile compared with lowest, dose-response meta-analysis, subgroup analysis, sensitivity analyses and assessment of publication bias are detailed in the methods (Additional file 3).	6, 7
Provision of appropriate tables and graphics	We provided 4 tables (Additional file 2, 3 & 4)..	25
	**Reporting of results should include**	
Graph summarizing individual study estimates and overall estimate	See meta-analysis results of highest exposure quantile vs. lowest and dose-response trend (Figure 2, 3 & 4)	9, 10
Table giving descriptive information for each study included	See characteristics of the included studies (Table 1)	8, 9

Results of sensitivity testing	See results of sensitivity analysis and subgroup analysis. (Table 2 & 3)	10, 11
Indication of statistical uncertainty of findings	95% confidence intervals were presented with all summary estimates, I^2 values, results of sensitivity analyses , publication analysis and counter-enhanced funnel plot.(Additional file 4)	11
	**Reporting of discussion should include**	
Quantitative assessment of bias	Q test and I^2 statistic indicated moderate heterogeneity in strengths of the relationship due to most common biases in observational studies. Evaluation of heterogeneity is a crucial part in the present meta-analysis.	12, 13 &14
Justification for exclusion	We performed sensitivity analysis omitting a study to reduce the influence of potential selective bias on the overall estimate, in view of probable selection bias..	14
Assessment of quality of included studies	We discussed the results of the sensitivity analyses, and potential reasons for the observed heterogeneity.	13
	**Reporting of conclusions should include**	
Consideration of alternative explanations for observed results	There was discrepancy between diet and serum ratio of n-3/n-6 associated with BC risk led to explaining the conclusion cautiously.	15
Generalization of the conclusions	The present meta-analysis provides important public health significances for prevention and control of BC	15
Guidelines for future research	Tissue biomarker of n-3/n-6 ratio and LC n-3 PUFA or ALA supplementation should be more attached importance to BC risk.	15
Disclosure of funding source	See acknowledgement	16
