# Supplementary material for: Ratio of n-3/n-6 PUFAs and risk of breast cancer: a meta-analysis of 274135 adult females from 11 independent prospective studies
Source: BMC Cancer. 2014 Feb 18;14:105. doi: 10.1186/1471-2407-14-105 (PMC4016587; doi:10.1186/1471-2407-14-105)
Supplement: Additional file 2 — Completed data extraction from eligible original studies. Information as to how the available data were extracted by using the standard extraction forms. [file 1471-2407-14-105-S2.pdf]

Ⅰ data of serum

Vatten, et al 1993

**Participants**	Cases selection: 87 developed breast cancer cases serum samples;<br>Controls selection: 235 controls selected by 3 inclusion criteria;<br>Participants Source: from serum bank in Norway<br>Year of birth: 1932 vs. 1932 (P_{50})
**Exposure**	Exposure factors: fatty acids level in serum phospholipids<br>Subgroup factor：Menopause state (55y)<br>Measure method：gas-liquid chromatography in cases and controls<br>Blind performance: the disease state of all serum samples blinded as a whole.
**Comparability**	Matching in case vs. control: age distribution, menopause state, region, storage and measure of serum.<br>Other base line data: no description<br>Group comparability: case/control,87/235;<br>Subgroup comparability: premenopausal (65/195) vs. postmenopausal (22/40)
**Outcomes**	Accept participants: 87/235<br>Measurable outcomes(mean ± SD, mg/l):<br>n-3PUFAs(ALA,EPA,DPA, and DHA),n-6PUFAs, and n-3/n-6 ratio;<br>Estimation of Risk: RR<br>Covariates and stratification: not adjusted covariates for no detailed information;<br>Available outcomes: RR of n-3/n-6 ratio in premenopausal (65/195)<br>highest quartile (297) compared with lowest (225) quartile: H vs. L<br>18/49 vs. 18/49 (H = 0.14) vs. (L = 0.14) exposure of serum n-3/n-6 ratio: RR = 1.0 (1.0-1.0);<br>12/52 vs. 18/49 (H = 0.19) vs. (L = 0.14): RR = 0.6(0.3 -1.4)<br>18/46 vs. 18/49 (H = 0.24) vs. (L= 0.14): RR = 1.1 (0.5 - 2.3)<br>17/48 vs. 18/49 (H = 0.36) vs. (L = 0.14): RR = 1.0 (0.4-2.1)
**Notes**	No report BMI and baseline data<br>Not adjusted covariates and explained<br>No estimation of risk in premenopausal and total case/control study<br>No clear diagnose method

Chajes, et al 1999

**Participants**	Participants Source: collected in 3 ongoing cohort studies of monitoring of trends and cardiovascular disease study (MONICA) in Sweden;<br>Cases selection: developed breast cancer in cohort studies;<br>Controls selection: a sub-set of cohort members who did not;<br>Definite inclusion and exclusion criteria;<br>Participants number: case vs. control (624,208/416)<br>participation rate: 85% and reasoned;<br>Age: case vs. control mean (55y)<br>Baseline data: Age at menarche (years), Parity, Age at first full-term pregnancy, Lactation (months), age at menopause, weight, height and BMI;
**Exposure**	Exposure factors: fatty acids in serum phospholipids;<br>Measure method：gas chromatography in cases and controls<br>Blind performance: no description
**Comparability**	Matching in case vs. control: age distribution, menopause state, region, age of blood sample and baseline variables (P > 0.05);<br>Group comparability: case vs. control (584, 196/388),
**Outcomes**	Accept subjects: 196/388<br>Follow-up time:10 years;<br>Measurable outcomes: (mean+ range) percentage<br>n-6 PUFAs, n-3PUFAs and ratio of n-3/n-6 PUFAs;<br>Estimation of Risk: RRs and 95% confidence intervals (CIs)<br>Covariates and stratification:<br>Adjusted for age at menarche, age at first full-term pregnancy, number of children, use of hormone-replacement therapy, height and weight;<br>Available outcomes: RRs of EPA/AA ratio in serum (H vs. L)<br>0.045 (< 0.09) vs. 0.045 (< 0.09), RRs = 1.0 (1.0–1.0);<br>0.135 (0.09 – 0.175) vs. 0.045 (< 0.09), RRs = 1.46 (0.75–2.83)<br>0.225 (0.175 – 0.275) vs. 0.045 (< 0.09), RRs = 1.52 (0.72 – 3.24);<br>0.315 (> 0.275) vs. 0.045 (< 0.09), RRs = 0.88 (0.42 – 1.86)
**Notes**	Blind performance: no description<br>Data of quantile exposure cutoff: no description

Saadatian-Elahi, et al 2002

**Participants**	Cohort Source: university women enrolled in cohort study of hormones, diet, and cancer in France (NYUWHS);<br>Inclusion and exclusion criteria: definite<br>Participants number: no description (age: 34-65)<br>Cases ascertainment: 197 cases of BC within NYUWHS<br>Controls ascertainment: same cohort members without BC matching 1:1 for case;<br>Diagnosis: by clinical interview and pathological documents;<br>Baseline data: height, Weight, body mass index, nulliparous, history of benign breast disease, family history of BC and reproductive variables;<br>● P > 0.05 (pared t test): age at menarche, age at first, full-term birth, age at menopause, and body mass index;<br>● P < 0.05: Other variables adjusted by conditional logistic regression
**Exposure**	Exposure ascertainment:<br>draw venous blood, fatty acids in serum phospholipids;<br>Measure method：gas-liquid chromatography in cases and controls<br>Blind performance: no description
**Comparability**	Matching in case vs. control: matched a case by age at recruitment (± 3 months), menopausal status at baseline (pre- or postmenopausal);<br>Group comparability: case vs. control (197/197)<br>Compounders adjusted:<br>adjusted by family history, age at first full term birth, cholesterol, and history of treatment for benign breast conditions<br>Stratification analysis: by menopause (pre- or postmenopausal)
**Outcomes**	Accepted subjects: case vs. control (197/197);<br>Follow-up time: 4.3 years (average); Follow-up rate: 95%<br>Main measurable outcomes: % of total FAs, mean (SD)<br>n-6 PUFAs, n-3 PUFAs (ALA, EPA, DPA and DHA) and ratio<br>Estimation of Risk: RRs and 95% confidence intervals (CIs)<br>Available outcomes: RRs and CIs (H quartile compared with L quartile)<br>ratio of n-3/n-6 PUFAs<br>● Pre-: H quartile compared with L quartile<br>< 0.08: RRs =1.00 (1.00–1.00), 0.08-0.16: RRs = 0.52 (0.18–1.47),<br>0.16-0.24: RRs = 0.47 (0.17–1.26), > 0.24: RRs = 1 0.60 (0.24–1.54)<br>● Post-: H quartile compared with L quartile<br>< 0.08: RRs =1.00 (1.00–1.00), 0.08-0.16: RRs = 0.52 (0.18–1.47),<br>0.16-0.24: RRs = 0.47 (0.17–1.26), > 0.24: RRs = 1 0.60 (0.24–1.54)
**Notes**	

Chajes, et al 2008

Participants	Cohort Source: female members of a national health insurance scheme covering teachers in the French education system and their spouses of the E3N cohort.<br>98,995 female volunteers aged 40–65 years,<br>Inclusion and exclusion criteria: definite<br>Participants number: 1152 (384/768) women (pre- and post-)<br>Cases selection: BC cases diagnosed by medical records (363);<br>Controls selection: (702) matched to each case by some factors (1:2);<br>Participation rate: 81%;<br>Baseline data:<br>● BMI, Age, age at menopause and smoking, $P > 0.05$;<br>● Age at first birth and parity, use of menopausal hormones and familial history: $P < 0.05$;
Exposure	Exposure factors: fatty acids level in serum phospholipids;<br>Measure method: gas chromatography in cases and controls;<br>Blind performance: no description
Comparability	Matching in case vs. control:<br>age, menopausal status (pre- or postmenopausal) at blood collection, fasting status (yes or no) at blood collection, study center (40 centers), and date of blood collection (same year) to cases with a 1: 2 ratio;<br>Group comparability: case/control, 384/768;<br>Compounders adjusted:<br>● Covariates: adjusting for body mass index, alcohol consumption, height, menopausal hormone use, educational level, parity, family history of breast cancer, and history of benign breast disease;
Outcomes	Accept participants: case vs. control (363/702)<br>Follow-up time:7 years<br>Follow-up rate: no description<br>Measurable outcomes (percentage of tFAs): % FAs, mean (SD)<br>n-3 PUFAs (ALA, EPA, DPA and DHA), n-6 PUFAs and ratio of n-6/n-3 PUFAs;<br>Available outcomes : ratio of n-6/n-3 PUFAs in serum phospholipids, RRs and CIs of high categories compared with lowest<br>Q_1, RRs = 1.00 (1.00, 1.00); Q_2: RRs = 0.95 (0.63, 1.44);<br>Q_3: RRs = 0.86 (0.56, 1.33); Q_4: RRs = 1.03 (0.67, 1.56);<br>Q_5: RRs = 0.76 (0.48, 1.20)
Notes	No performance of blindness<br>Data of quantile exposure cutoff: no description

Takata, et al 2009

**Participants**	Cohort Source: heavy cigarette smokers and asbestos-exposed workers postmenopausal women (age: 50-60y) from β-Carotene and Retinol Efficacy Trial (CARET) Cohort study in USA ;<br>Inclusion and exclusion criteria: definite<br>Participants number: menopause women<br>Cases selection: BC cases diagnosed by pathology reports;<br>Controls selection: matched to each case by some factors;<br>Participation rate: follow-up rate in this study was about 96%;<br>Definite Inclusion and exclusion criteria;<br>Baseline data:<br>● BMI, Age, Education, and total caloric intake, P > 0.05;<br>● Average age at enrollment (years): 58.6(5.4) vs. 58.6 (5.1), P > 0.05;<br>● Smoking and alcohol consumption, P < 0.05;
**Exposure**	Exposure factors: fatty acids level in serum phospholipids;<br>Measure method：gas chromatography in cases and controls;<br>Blind performance: no description
**Comparability**	Matching in case vs. control: age at enrollment, race, study center and year of enrollment to cases with a 1: 2 ratio;<br>Group comparability: case/control,130/257;<br>Compounders adjusted:<br>● Covariates: adjusting for all matching criteria (age, study center, and year of the enrollment) as well as intervention arm, smoking status at baseline and at blood draw (current vs. former smokers), BMI, and alcohol use;<br>● Stratification analysis: subgroups by smoking status;
**Outcomes**	Accept participants: case vs. control (103/309)<br>Follow-up time: 3 years; Follow-up rate: 96%<br>Measurable outcomes (weighted percentage of TFAs): % FAs, P50 (P25～P75) n-3 PUFAs(ALA, EPA, DPA and DHA), n-6 PUFAs and n-3/n-6 ratio;<br>Estimation of Risk: RRs and 95% confidence intervals (CIs)<br>Available outcomes : n-3 PUFAs in serum phospholipids, ORs and CIs<br>● Q_1:< 0.11, RRs =1.00(1.00–1.00);<br>● Q_2:0.11-0.12, RRs = 0.75 (0.41–1.37);<br>● Q_3:0.12-0.15, RRs = 0.60 (0.32–1.14)<br>● Q_4:> 0.15, RRs = 0.74 (0.40–1.36);
**Notes**	No performance of blindness

Ⅱ data of diet

Wirfalt et al 2002

**Participants**	Cohort Source: a cohort of 74,138 individuals(men n = 11,063; women n = 17,035) in the city of Malmo in Sweden;<br>Participants number: 12,803 women (age ≥ 50 years)<br>Cases ascertainment: 249 cases verified from cohort by record linkage with the Swedish Cancer Registry;<br>Controls ascertainment: from women without breast cancer (n = 12,039) at the time of study entry and during follow-up;<br>Inclusion and exclusion criteria: definite<br>Diagnosis: by record linkage with the Swedish Cancer Registry;<br>Baseline data:<br>● age at menarche, level of education and exercise, waist circumference energy, height, age at first childbirth, intake of alcohol, smoking and education level: P > 0.05;,<br>● ,Body mass index and current HRT users: P < 0.05;
**Exposure**	Exposure ascertainment: fatty acids in dietary PUFAs;<br>Measure method: a structured food frequency questionnaire (FFQ) in cases and control women;<br>Blind performance: no description
**Comparability**	Matching in case vs. control: 1:3;<br>Group comparability: case vs. control (237/673), some subjects were excluded and reasoned;<br>Compounders adjusted: Past food habit change, energy intake, BMI, height, waist circumference, age at birth of first child, current hormone therapy, alcohol habits, and educational status;
**Outcomes**	Accept subjects: case vs. control (237/673) ;<br>Follow-up time: 3-8 years<br>Main measurable outcomes: dietary fatty acids (g/d)<br>n-6 PUFAs, n-3PUFAsand n-3/n-6 ratio;<br>Estimation of Risk: RRs and 95% confidence intervals (CIs)<br>0.15 vs. 0.15, RR = 1(1,1); 0.18 vs. 0.15, RRs = 0.77 (0.47, 1.24);<br>0.20 vs. 0.15, RRs = 0.91 (0.56, 1.46); 0.24 vs. 0.15, RRs = 0.77 (0.47,1.26);<br>0.33 vs. 0.15,, RRs = 0.66 (0.41,1.08),
**Notes**	Blind performance: no description;

Wakai et al, 2005

**Participants**	Cohort Source: the Japan Collaborative Cohort (JACC) Study;<br>Inclusion and exclusion criteria: definite;<br>Participants number: 26,291 women of aged 40–79 years;<br>Cases ascertainment:129 incident cases from cohort;<br>Diagnosis: by means of a link-age with the records of population-based cancer registries;<br>Baseline data: Age (years), Education beyond high school, Family history of breast cancer in mother and/or sisters, Age at menarche (years), Menopause, Age at menopause (years), Age at first birth (years),<br>Parity, Ever used exogenous female hormones, Alcohol consumption, Smoking and so on.
**Exposure**	Exposure ascertainment: dietary fatty acids and n-6/n-3 ratio (% energy);<br>Measure method：FFQ(40 food items)<br>Blind performance: no description
**Comparability**	Group comparability: case vs. cohort (129/36,035)<br>Compounders adjusted: Age, study area, educational level, family history of breast cancer, age at menarche, age at menopause, age at first birth, parity, use of exogenous female hormones, alcohol consumption, smoking, consumption of green leafy vegetables, daily walking, height,<br>body mass index, and total energy intake
**Outcomes**	Accept subjects: case vs. control (129/26291) and reasoned;<br>Follow-up time: 7.6 years (average);<br>Lose of follow-up rate:2.70% and reasoned;<br>Main measurable outcomes: dietary fatty acids and ratio of n-6/n-3 PUFAs<br>Estimation of Risk: RR and 95% confidence intervals (CIs)<br>Available outcomes: multivariate RRs and CIs (Highest vs. lowest quartile)<br>Ratio of n-6/n-3 PUFAs: < 3.25, RRs = 1.00 (1.00-1.00);<br>3.25 – 3.90, RRs = 0.95 (0.55-1.62);<br>3.91– 4.60, RRs = 1.57 (0.97–2.56);<br>⩾4.61, RRs = 1.31 (0.78–2.19);
**Notes**	Blind performance: no description

Thiebaut, et al.2009

**Participants**	Cohort Source: The European Prospective Investigation into Cancer and Nutrition (EPIC);<br>Inclusion and exclusion criteria: definite;<br>Participants number: 98,995 women volunteers aged 40-65 years;<br>Cases ascertainment: 1,864 incident breast cancer cases from cohort;<br>Diagnosis: self-reported cases and confirmed by a pathology report (96.6%).;<br>Baseline data: educational level, reproductive history, history<br>of benign breast diseases, familial history of breast cancer and hormonal treatments and so on.
**Exposure**	Exposure ascertainment: dietary fatty acids and n-6/n-3 ratio (% energy);<br>Measure method：FFQ (208 food items)<br>Blind performance: no description
**Comparability**	Group comparability: case vs.cohort (1,864 /73,034)<br>Compounders adjusted: Age, nonalcohol energy and ethanol intakes, smoking history, history of benign breast disease and breast cancer, age at menarche, parity, body mass index, menopausal status, age at menopause<br>and use of menopausal hormone treatment
**Outcomes**	Accept subjects: case vs. control (1650/56007) and reasoned;<br>Follow-up time: 8.0 years (average);<br>Response rate: 81.10% and reasoned;<br>Main measurable outcomes: dietary fatty acids and ratio of n-6/n-3 PUFAs<br>Estimation of Risk: RR and 95% confidence intervals (CIs)<br>Available outcomes: multivariate RRs and CIs (Highest vs. lowest quartile)<br>Ratio of n-6/n-3 PUFAs:<br>Q_1 = 5.48, RRs = 1.00 (1.00, 1.00);<br>Q_2 = 7.33, RRs = 1.06 (0.91, 1.23);<br>Q_3 = 8.95, RRs = 1.04 (0.89, 1.21);<br>Q_4 = 10.91, RRs = 0.93 (0.80, 1.09);<br>Q_5 = 14.76, RRs = 0.97 (0.83, 1.14).
**Notes**	Blind performance: no description

Murff, et al.2011

**Participants**	Cohort Source: Shanghai Women Health Study (SWHS) cohort study;<br>Inclusion and exclusion criteria: definite;<br>Participants number: 74,942 women aged 40–70 years from seven urban communities in Shanghai;<br>Cases ascertainment: 712incident breast cancer cases from cohort;<br>Diagnosis: by medical charts from the diagnostic hospital;<br>Baseline data: Age at baseline (years), Family history of breast cancer, Education, Smoking, Age at menarche (years), Age at menopause, Use of hormone replacement therapy, Age at first pregnancy (years),<br>Body mass index, Waist-to-hip ratio, Total energy intake (kcal/day) and so on.
**Exposure**	Exposure ascertainment: dietary fatty acids and n-6/n-3 ratio (g/d);<br>Measure method：FFQ<br>Blind performance: no description
**Comparability**	Group comparability: case vs. cohort (712/71,859)<br>Compounders adjusted: Age, body mass index, total energy, family history of breast cancer, alcohol use, tobac co use, education, hormone replacement therapy, personal history of diabetes, menopausal status, age at menopause, age at menarche, parity, age at first pregnancy, level of physical activity, red meat intake, fish intake and vitamin E intake
**Outcomes**	Accept subjects: case vs. control (712/72,571) and reasoned;<br>Follow-up time: 8.0 years (average);<br>Response rate: 99.98% and reasoned;<br>Main measurable outcomes: dietary fatty acids and ratio of n-6/n-3 PUFAs<br>Estimation of Risk: RR and 95% confidence intervals (CIs)<br>Available outcomes: multivariate RRs and CIs (Highest vs. lowest quartile)<br>Ratio of n-6/n-3 PUFAs:<br>Q_1 = 5.18, RRs = 1.00 (1.00, 1.00);<br>Q_2 = 5.83, RRs = 0.93 (0.73–1.19);<br>Q_3 = 6.29, RRs = 0.98 (0.76–1.26);<br>Q_4 = 6.78, RRs =0.90 (0.69–1.18);<br>Q_5 = 7.64, RRs = 1.02 (0.77–1.34).
**Notes**	Blind performance: no description

Park,et al.2012

**Participants**	Cohort Source: The Multiethnic Cohort Study (MEC);<br>Inclusion and exclusion criteria: definite;<br>Participants number: 99,800 postmenopausal women of age > 55 years;<br>Cases ascertainment: 3,885 incident invasive cases were identified;<br>Diagnosis: by linkage of the cohort to the Surveillance, Epidemiology, and End Results (SEER) cancer registries covering Hawaii and California;<br>Baseline data: Age at cohort entry, Ethnicity, Family history of breast cancer, Education, BMI at cohort entry,<br>Smoking, Age at menarche, Age at first live birth, Number of children, Age at and type of menopause, Oophorectomy, Hysterectomy, use of hormone replacement therapy (ever and never users), follow-up period, family history of breast cancer (yes and no), smoking status and son.
**Exposure**	Exposure ascertainment: dietary fatty acids and n-6/n-3 ratio (g/d);<br>Measure method：FFQ (180 food items)<br>Blind performance: no description
**Comparability**	Group comparability: case vs. cohort (3,885/85,089)<br>Compounders adjusted: Age at cohort entry, ethnicity, family history of breast cancer, education, BMI, age at menarche, age at first l ive birth, number of children, age at and type of menopause, hormone replacement therapy, smoking status, energy intake, and alcohol.
**Outcomes**	Accept subjects: case vs. control (3,885/85,089) and reasoned;<br>Follow-up time: 12.4 years (average);<br>Response rate: 91.30% and reasoned;<br>Main measurable outcomes: dietary fatty acids and ratio of n-6/n-3 PUFAs<br>Estimation of Risk: RR and 95% confidence intervals (CIs)<br>Available outcomes: multivariate RRs and CIs (Highest vs. lowest quartile)<br>Ratio of n-6/n-3 PUFAs:<br>< 7.6, RRs = 1.00 (1.00, 1.00);<br>7.6 – 8.3, RRs = 1.12 (1.02–1.24);<br>8.3 – 8.8, RRs = 1.09 (0.98–1.20);<br>8.8 - 9.6, RRs = 1.01 (0.91–1.12);<br>> 9.6, RRs = 1.10 (0.99–1.22).
**Notes**	Blind performance: no description

Sczaniecka et al, 2012

**Participants**	Cohort Source: female members of the Vitamins And Lifestyle (VITAL) Cohort;<br>Inclusion and exclusion criteria: definite;<br>Participants number: 168,953 women of age 50-76 years;<br>Cases ascertainment: 772 incident invasive cases were identified;<br>Diagnosis: through all area hospitals, offices of pathologists, oncologists, and radiotherapists, and from state death certificates;<br>Baseline data: Age at baseline, Race, Age at first birth, First-degree relatives with breast cancer, BMI, Physical activity, Alcohol intake, Total energy intake.
**Exposure**	Exposure ascertainment: dietary fatty acids and n-3/n-6 ratio (g/d);<br>Measure method：FFQ (120 food items)<br>Blind performance: no description
**Comparability**	Group comparability: case vs. cohort (772/40,337)<br>Compounders adjusted: Age, race, education, height, body mass index, age at menarche, age at first birth, age at menopause, history of hysterectomy, years of combined hormone therapy, years of estrogen hormone therapy, family history of breast cancer, mammography, history of benign breast biopsy, regular use of no steroidal anti-inflammatory drugs, exercise, alcohol consumption, vegetable intake, fruit intake, and total energy intake.
**Outcomes**	Accept subjects: case vs. control (772/ 30,252) and reasoned;<br>Follow-up time: 6.0 years (average);<br>Response rate: 87.80% and reasoned;<br>Main measurable outcomes: dietary fatty acids and ratio of n-3/n-6 PUFAs<br>Estimation of Risk: RR and 95% confidence intervals (CIs)<br>Available outcomes: multivariate RRs and CIs (Highest vs. lowest quartile)<br>Ratio of n-6/n-3 PUFAs:<br>< 0.005, RRs = 1.00 (1.00, 1.00);<br>0.005 - 0.01, RRs = 1.03 (0.81, 1.29);<br>0.01 - 0.02, RRs = 1.04 (0.83, 1.31);<br>0.02 - 0.03, RRs = 1.02 (0.81, 1.30);<br>≥ 0.03, RRs = 0.84 (0.65, 1.09).
**Notes**	Blind performance: no description
