# Supplementary material for: Ratio of n-3/n-6 PUFAs and risk of breast cancer: a meta-analysis of 274135 adult females from 11 independent prospective studies
Source: BMC Cancer. 2014 Feb 18;14:105. doi: 10.1186/1471-2407-14-105 (PMC4016587; doi:10.1186/1471-2407-14-105)
Supplement: Additional file 3: Table S1 — Details on data synthesis and analysis in this meta-analysis. Studies excluded after scrutiny with reasons for exclusion. Table S2. Study quality assessment of included prospective studies by Newcastle-Ottawa Scale. Figure S1. Dose–response trend between serum PL ratio of n-3/n-6 PUFAs and breast cancer risk among USA females. Figure S2. Contour-enhanced funnel plots for association of ratio of n-3/n-6 PUFA with BC risk. [file 1471-2407-14-105-S3.doc]

**Details of data synthesis:**

- **Meta-analysis for the highest quantile vs. lowest or reference**

For a study without RR for n-3/n-6 or n-6/n-3 ratio but providing that for ALA (18:3n-3,linolenic acid)/AA (20:4n-6, arachidonic acid), EPA (20:5n-3, eicosapentaenoic acid)/AA, DPA (22:5n-3, [docosapentaenoic](app:ds:docosapentaenoic) [acid](app:ds:acid))/AA and DHA (22:6n-3, docosahexaenoic acid)/AA separately, these RRs were combined to represent the pooled RR for n-3/n-6 ratio exposure in this study; if the original reference only provided one of ALA/AA, EPA/AA, DPA/AA and DHA/AA , the RR was used to approximately represent RR for the association between n-3/n-6 ratio and breast cancer risk in the original reference. Finally, the pooled RR of all included studies has been estimated by random effect model in present meta-analysis.

- **Meta-analysis of dose-response**

Studies with 3 or more exposure categories were included in summarized dose-response analysis. Midpoint of upper and lower boundaries was taken as the dose of the category if the study only reported the range of serum or dietary n-3/n-6 ratio; if the highest category was open-ended, its dose was regarded as the same amplitude as the preceding one plus half of highest boundary; if the lowest or reference category was open-ended, midpoint of lowest boundary and zero was taken as the dose of lowest category. For dietary ratio of n-3/n-6 PUFAs as exposure, dose-response trend estimate was performed by means of Generalized Least Squares (GLS) described by Greenland and Orsini et al. For serum ratio of n-3/n-6 PUFAs as exposure, dose-response trend estimate was performed by means of Variance-weighted least squares regression (WLS), in view of number of cases from exposure quantile was not available.

**Table S1. Studies excluded after scrutiny with reasons for exclusion**

| **Reference** | **Reasons for exclusion** |
| --- | --- |
| 1. Bougnoux P, Koscielny S, Chajes V, et al. Alpha-linolenic acid content of adipose breast tissue: A host determinant of the risk of early metastasis in breast cancer. Br J Cancer. 1994; 70(2):330-334. | Providing RR for BC metastasis risk associated with n-3 PUFAs, but no data of BC occurrence risk associated with n-3/n-6 ratio.  However, BC incidence was taken as outcome variable in our present study. |
| 2. Petrek JA, Hudgins LC, Ho M, et al. Fatty acid composition of adipose tissue, an indication of dietary fatty acids, and breast cancer prognosis. Journal of Clinical Oncology. 1997;15(4):1377-1384. | Evaluating RR for BC prognosis risk associated with n-3 PUFAs and n-6 PUFAs in adipose tissue, no data of n-3/n-6 ratio.  However, BC incidence was taken as outcome variable in our present study. |
| 3. Holmes MD, Hunter DJ, Colditz GA, et al. Association of dietary intake of fat and fatty acids with risk of breast cancer. JAMA-Journal of the American Medical Association. 1999; 281(10):914-920. | Only providing RR for association between BC risk and per 0.1% energy increment of dietary n-3 PUFAs |
| 4. Kobayashi M, Sasaki S, Hamada GS, et al. Serum n-3 fatty acids, fish consumption and cancer mortality in six Japanese populations in Japan and brazil. Japanese Journal of Cancer Research. 1999; 90(9):914-921. | Providing serum fatty acid composition in Japanese men in six Regions in Japan and Brazil, and cancer mortality as outcome variable. |
| 5. Pala V, Krogh V, Muti P, et al. Erythrocyte membrane fatty acids and subsequent breast cancer: a prospective Italian study. J Natl Cancer Inst. 2001 Jul 18;93(14):1088-95. | Only providing the adjusted OR for tissue n-3 PUFAs related to BC risk among postmenopausal women. |
| 6. Voorrips LE, Brants HA, Kardinaal AF, Hiddink GJ, van den Brandt PA, Goldbohm RA. Intake of conjugated linoleic acid, fat, and other fatty acids in relation to postmenopausal breast cancer: the Netherlands Cohort Study on Diet and Cancer. Am J Clin Nutr. 2002 Oct;76(4):873-82. | Only providing the data of dietary n-3 and n-6 PUFAs associated with BC risk, but no data of n-3/n-6 ratio. |
| 7. Rissanen H, Knekt P, Jarvinen R, et al. Serum fatty acids and breast cancer incidence.*NutrCancer*.2003;45(2):168-175. | Only providing RR for n-6 PUFAs in serum PL associated with BC risk. |
| 8. Gago-Dominguez M, Yuan JM, Sun CL, Lee HP, Yu MC. Opposing effects of dietary n-3 and n-6 fatty acids on mammary carcinogenesis: The Singapore Chinese Health Study. British Journal of Cancer. 2003;89(9):1686-92. | Providing the adjusted OR between dietary n-3 and n-6 associated with BC risk, no data of n-3/n-6 ratio. |
| 9. Cho E, Spiegelman D, Hunter DJ, et al. Premenopausal fat intake and risk of breast cancer. J Natl Cancer Inst. 2003 Jul 16;95(14):1079-85. | The study was prospective cohort study design, but it only provided RR for long chain n-3 PUFAs related to BC risk |
| 10. Wirfalt E, Vessby B, Mattisson I, et al. No relations between breast cancer risk and fatty acids of erythrocyte membranes in postmenopausal women of the Malmo diet cancer cohort (Sweden). European journal of clinical nutrition. 2004;58(5):761-770. | Providing the adjusted RR for association between per 0.1 unit of n-3/n-6 ratio in erythrocyte membrane and BC risk.  However, we only included the data from serum phospholipids. |
| 11. White E, Patterson RE, Kristal AR, et al. VITamins and Lifestyle Cohort Study: Study Design and Characteristics of Supplement Users. American Journal of Epidemiology. 2004;159(1):83-93. | No data of n-3, n-6 and n-3/n-6 ratio related to BC risk. |
| 12. 1 Engeset D, Alsaker E, Lund E, et al. Fish consumption and breast cancer risk. The European Prospective Investigation into Cancer and Nutrition (EPIC). International Journal of Cancer. 2006;119(1):175-82. | Only providing RR for fish intake and BC risk. |
| 13. Kim EH, Willett WC, Colditz GA, et al. Dietary fat and risk of postmenopausal breast cancer in a 20-year follow-up. Am J Epidemiol. 2006 Nov 15;164(10):990-7. | Only providing the data of 18: 3n-3 and n-3 PUFAs intake related to BC risk, but no providing the data of n-6 and n-3/n-6 ratio. |
| 14.Touvier M, Kesse E, Volatier JL, Clavel-Chapelon F, Boutron-Ruault MC. Dietary and cancer-related behaviors of vitamin/mineral dietary supplement users in a large cohort of French women. European Journal of Nutrition. 2006 Jun;45(4):205-14. | Providing RR for association between dietary n-3 and n-6 PUFAs supplement and BC risk. |
| 15. Wu AH, Koh WP, Wang R, Lee HP, Yu MC. Soy intake and breast cancer risk in Singapore Chinese Health Study. British Journal of Cancer. 2008;99(1):196-200. | Only providing the data of soy isoflavones related to BC risk. |
| 16. Schulz M, Hoffmann K, Weikert C, Nothlings U, Schulze MB, Boeing H. Identification of a dietary pattern characterized by high-fat food choices associated with increased risk of breast cancer: The European Prospective Investigation into Cancer and Nutrition (EPIC)-Potsdam Study. British Journal of Nutrition. 2008;100(5):942-6. | No providing RR for dietary n-3, n-6 and n-3/n-6 ratio related to BC risk. Only providing adjusted hazard ratios (HR) and 95 % CI for the association between the simplified food pattern score and risk of breast cancer. |
| 17. Sonestedt E, Ericson U, Gullberg B, Skog K, Olsson H, Wirfalt E. Do both heterocyclic amines and omega-6 polyunsaturated fatty acids contribute to the incidence of breast cancer in postmenopausal women of the Malmo diet and cancer cohort? International Journal of Cancer. 2008;123(7):1637-43. | Providing HR for association between dietary n-6 PUFAs, heterocyclic amines (HAs) and BC risk. |
| 18. Witt PM, Christensen JH, Schmidt EB, et al. Marine n-3 polyunsaturated fatty acids in adipose tissue and breast cancer risk: a case-cohort study from Denmark. Cancer Causes Control. 2009 Nov;20(9):1715-21. | Only providing the data of long chain n-3 PUFAs related to BC risk. |
| 19. Hedelin M, Lof M, Olsson M, et al. Dietary intake of fish, omega-3, omega-6 polyunsaturated fatty acids and vitamin D and the prevalence of psychotic-like symptoms in a cohort of 33 000 women from the general population. Bmc Psychiatry. 2010 May;10. | Providing relative risk of positive psychotic-like symptoms in relation to estimated dietary intake of fatty acids. |
| 20. Patterson RE, Flatt SW, Newman VA, et al. Marine fatty acid intake is associated with breast cancer prognosis. J Nutr. 2011 Feb;141(2):201-6. | Providing RR for relationshipi between dietary marine n-3 PUFAs and BC prognosis risk. However, BC incidence was taken as outcome variable in our present study. |
|  |  |

**Table S2. Study quality assessment of included prospective studies by Newcastle-Ottawa Scale (Stars).**

| Study design | **Selection(☆☆☆☆)** | | **Comparability(☆☆)** | **Exposure or Outcome (☆☆☆)** | | | **Stars** | | **Study quality** |
| --- | --- | --- | --- | --- | --- | --- | --- | --- | --- |
| **Cohort studies** | - Representativeness of the exposed cohort? ☆   2) Selection of the non exposed cohort? ☆  3) Evaluating exposure? ☆  4) Outcomes of interest were not present at study start? ☆ | | 1) Study controls for the most important factor? ☆  2) Study controls for any additional factors? ☆ | - How to ascertain outcome? ☆ a) Independent blindness   b) record linkage   - Follow-up till outcomes happened? ☆ - Adequacy of follow up? ☆ | | | ☆☆☆☆  ☆☆  ☆☆☆ | | High quality: 8-9stars  Moderate quality:  6-7stars  Low quality:1-5 stars |
| **Nested case-control** | 1) Adequate case definition? ☆  2) Representativeness of the cases? ☆  3) Community controls? ☆  4) Controls have no history of endpoint disease? ☆ | | 1) Study controls for the most important factor? ☆  2) Study controls for any additional factors? ☆ | 1. How to ascertain exposure?☆ a) By secure record b)Structured interview where blind to case/control status 2. Same method of ascertainment for both? ☆ 3. Same response rate for both? ☆ | | |
| **Included Cohort Studies** | | | | | | | | | |
| Wakai et al, 2005. (Japan) | 1) ☆; 2) ☆,  3) no statement, 4) ☆ | | 1) ☆, 2) ☆ | 1) ☆, 2) ☆, 3) ☆ | | | ☆☆☆  ☆☆  ☆☆☆ | | High |
| Thiebaut, et al.2009 (France) | 1) : teachers cohort; 2) ☆, 3) :no statement; 4) ☆ | | 1) ☆;, 2) ☆ | 1) ☆, 2) ☆, 3) ：no description | | | ☆☆  ☆☆  ☆☆ | | Moderate |
| Murff, et al.  2011.(China) | 1) ☆; 2) ☆, 3) ☆, 4) ☆ | | 1) ☆; 2) ☆ | 1) ☆, 2) ☆, 3) ☆ | | | ☆☆☆☆  ☆☆  ☆☆☆ | | High |
| Park, et al.  2012. (USA) | 1)☆, 2) ☆, 3) ☆, 4)☆ | | 1) ☆; 2) ☆ | 1) ☆, 2) ☆, 3) no statement | | | ☆☆☆☆  ☆☆  ☆☆ | | High |
| Sczaniecka, et al. 2012.  (USA) | 1) ☆, 2) ☆;  3) :no statement; 4) ☆; | | 1) ☆; 2) ☆ | 1) ☆, 2) ☆, 3) ☆ | | | ☆☆☆  ☆☆  ☆☆☆ | | High |
| **Included Nested Case-Control Studies** | | | | | | | | | |
| Vatten,et al  1993 (Norway) | 1)no statement, 2) ☆,  3) ☆, 4) ☆; | 1) ☆  2) : without covariates  adjusted | | | 1) ☆, 2) ☆, 3) ☆ | ☆☆☆  ☆  ☆☆☆ | | Moderate | |
| Chajes,et al  1999 (Sweden) | 1) :no mention, 2) ☆,  3) ☆ 4) :no mention | 1) ☆；2) ☆ | | | 1) ☆, 2) ☆, 3) ☆ | ☆☆  ☆☆  ☆☆☆ | | Moderate | |
| Saadatian-  Elahi, et al  2002 (USA) | 1) ☆ 2) ☆,  3) ☆, 4) ☆ | 1) ☆, 2) ☆ | | | 1) ☆, 2) ☆,  3) ☆ | ☆☆☆☆  ☆☆  ☆☆☆ | | High | |
| Chajes,et al  2008 (Sweden) | 1) ☆ 2) : teacher volunteers, 3) ☆, 4) ☆ | 1) ☆, 2) ☆ | | | 1) ☆, 2) ☆, 3) :no mention | ☆☆☆  ☆☆  ☆☆ | | Moderate | |
| Takata,et al  2009 (USA) | 1) ☆, 2) ☆,  3) ☆, 4) :no mention | 1) ☆, 2) ☆ | | | 1) ☆, 2) ☆,  3) ☆ | ☆☆☆  ☆☆  ☆☆☆ | | High | |
| Wirfalt, et al. 2002 (Sweden) | 1) :record linkage; 2) : higher education and white collar workers,  3) ☆, 4) ☆ | 1) ☆, 2) ☆ | | | 1) ☆, 2) ☆,  3) no statement | ☆☆  ☆☆  ☆☆ | | Moderate | |

**Footnotes**

Yes=☆, No=;

**Figure S1. Dose-response trend between serum PL ratio of n-3/n-6 PUFAs and breast cancer risk among USA females.**


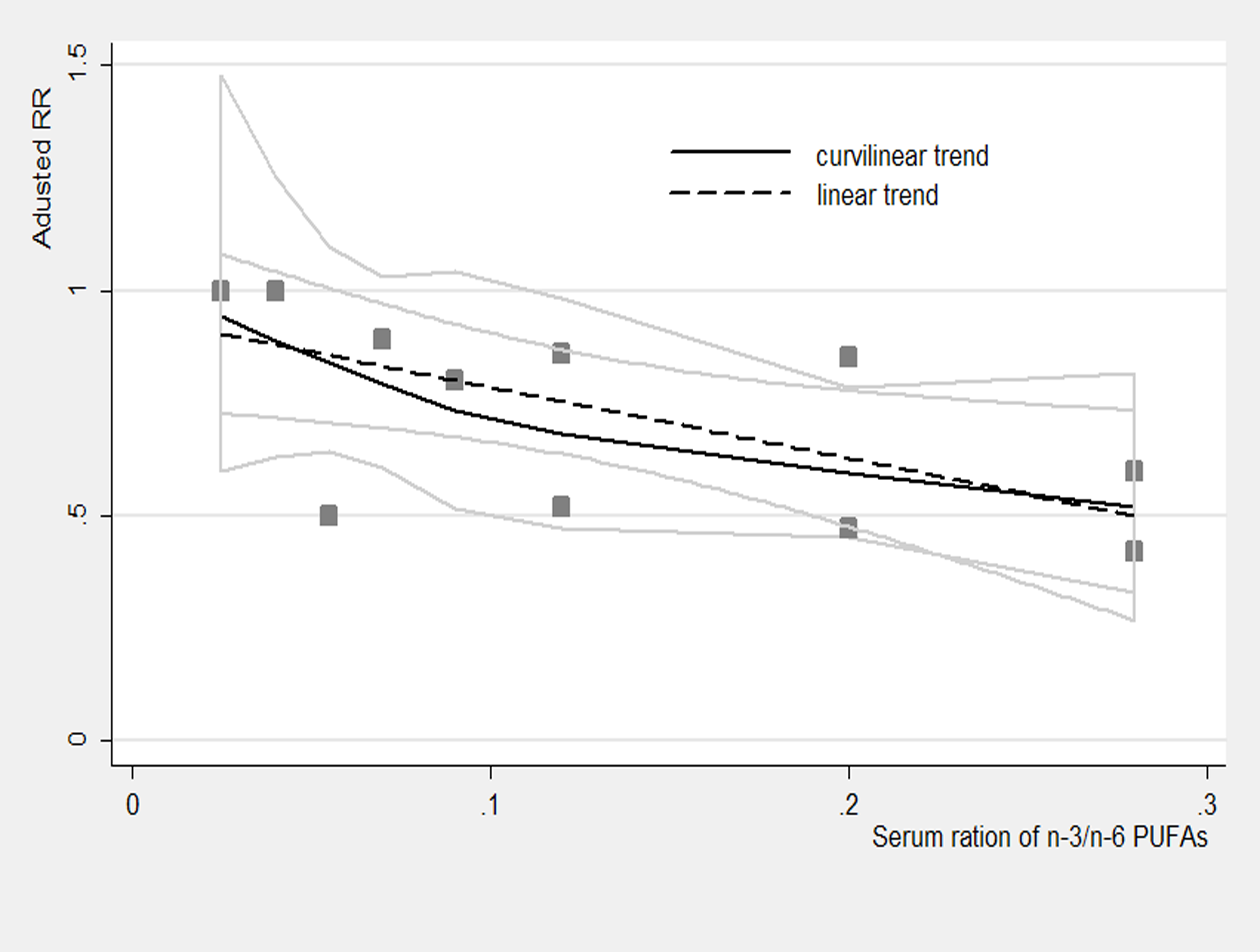


**Figure S2. Contour-enhanced funnel plots for association of intake n-3/n-6 ratio with breast cancer risk.**

**
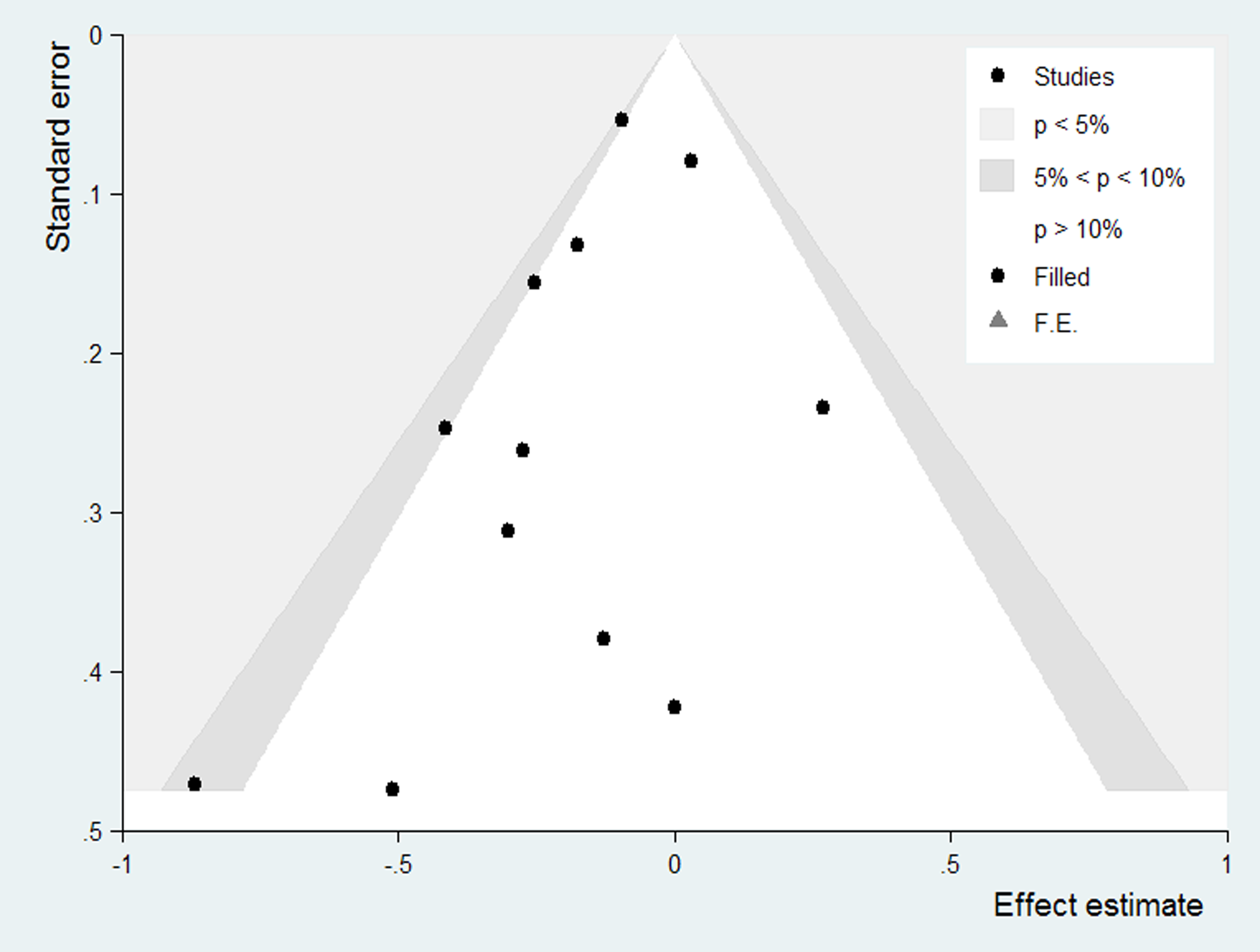
**
